# Water vapor transport observed at a coastal Mediterranean site during the summer of 2021 and compared with ERA5

**DOI:** 10.1038/s41598-026-36040-0

**Published:** 2026-02-14

**Authors:** Fabio Madonna, Ilaria Gandolfi, Yassmin Hesham Essa, Benedetto De Rosa, Simone Gagliardi, Domenico Madonna, Fabrizio Marra, Maria Assunta Menniti, Donato Summa, Emanuele Tramutola, Faezeh Karimian Saracks, Filomena Romano, Marco Rosoldi

**Affiliations:** 1 Department of Physics, University of Salerno, Fisciano, Italy; 2 Istituto di Metodologie per l’Analisi Ambientale (CNR-IMAA), Consiglio Nazionale delle Ricerche, Tito Scalo, Italy; 3 Institute for Atmospheric and Environmental Sciences, Goethe University Frankfurt (GUF-IAU), Frankfurt am Main, Germany; 4 Earth System Analysis, Potsdam Institute for Climate Impact Research (PIK), Member of the Leibniz Association, Potsdam, Germany; 5 Istituto Salesiano Sant’Antonio di Padova, Soverato, Italy; 6 Centro Studi e Ricerca Ambiente Marino (CESRAM), Soverato, Italy

**Keywords:** Mediterranean, Water vapor, Observations, Renalaysis, Extreme events, Climate sciences, Environmental sciences, Natural hazards

## Abstract

Extreme events in the Mediterranean have increased in frequency and intensity over the past decade, and this trend is expected to continue. Characterizing these events using reliable observational datasets is essential for understanding the processes driving their development. This study uses observational data collected during the Mediterranean Experiment for Sea Salt And Dust Ice Nuclei (MESSA-DIN) from July to September 2021, at the coastal site of Soverato, on the Ionian coast of Southern Italy, to characterize the intense water vapor transport over the Mediterranean basin during the severe summer of 2021. Furthermore, we compare the observations to the atmospheric reanalysis ERA5 and describe the synoptic environment over the study period. The results highlight the role of water vapor transport in weather systems and local atmospheric processes, underscoring the importance of high-resolution water vapor atmospheric profiling to enhance the accuracy of extreme rainfall and flood forecasting. While ERA5 performed well in capturing synoptic-scale patterns, it exhibited a dry bias of up to 3 g kg-1 compared to microwave radiometer retrievals in the 450–650 hPa vertical layer. This bias likely reflects limitations in the reanalysis representation of tropospheric moisture transport, particularly from dominant North African inflows, in addition to contributions from remote Atlantic pathways and local sources.

**Supplementary Information:**

The online version contains supplementary material available at 10.1038/s41598-026-36040-0.

## Introduction

The Mediterranean region often experiences significant water vapor transport, sustained by sea surface evaporation, particularly during summer, which is a significant source of moisture for both zonal and meridional transport of air masses^1^. Severe precipitation in the Mediterranean Basin depends on both remote and local sources of intense surface evaporation^2,3^. Transport of water vapor from the Atlantic, North Africa, and the surrounding regional seas, contributes to the elevated humidity observed in the troposphere, especially during the summer months, and plays a vital role in the region’s atmospheric and hydrological systems, shaping precipitation patterns, modulating extreme weather events, and influencing broader climate dynamics^4^. It also has profound effects on the regional climate, intensifying rainfall in some areas while prolonging droughts in others. Persistent water vapor advection also affects the regional radiative budget by enhancing surface radiation trapping, thereby amplifying the severity of heatwaves. A comprehensive understanding of these processes within the Mediterranean basin is therefore essential for improving predictions of extreme weather events, including the recurrent severe floods and heatwaves affecting the region^5^.

As a recognized climate change hotspot, the Mediterranean region is particularly sensitive to increase in atmospheric moisture, which may influence weather systems, including the intensification of extratropical cyclones. While recent studies report good agreement regarding the global climatology of water vapor transport, atmospheric reanalyses and climate models still exhibit systematic errors in the representation of water vapor in the boundary layer, often accompanied by compensating errors in the free troposphere^6–8^. This limitation is particularly pronounced in coastal regions, where small-scale processes such as convection, orography, turbulent mixing, and land–sea interactions are inherently challenging to resolve. Further regional-scale studies are therefore needed, with particular focus on areas where water vapor transport exerts a critical influence on extreme precipitation. Recent advances in observational programs have underscored the importance of accurately representing transport of atmospheric moisture in forecasting severe weather. For example, water vapor transport has been identified as a key ingredient in Mediterranean high-precipitation events (HPEs) and for improving short-term forecasts of precipitation timing and location in coastal mountainous regions^9^. Likewise, the lower tropospheric water vapor concentration profile plays a crucial role in enhancing such short-term predictions. Furthermore, previous studies have demonstrated that the initiation of convection in cloud-resolving models can be predicted with high reliability when water vapor distributions within and above the boundary layer are characterized with adequate vertical resolution and accuracy (e.g^10,11^).

During the summer of 2021, one of the warmest on record for Europe in recent decades^12^, several Mediterranean regions experienced severe soil moisture deficits. Southwestern Europe faced dramatic heatwaves during summer and early autumn, as reported in the European State of the Climate^13^. Recent studies indicate that the highest recorded temperatures were observed in southern Italy^14^. Dry conditions in the northern Mediterranean basin extended into northern Tunisia, while soil moisture levels in other parts of northern Africa, along the coastline, were near or exceeding the climatological values^15^.

This study investigates the water vapor transport observed at a coastal site during the summer of 2021, using a combination of ground-based measurements from the mobile facility of the Atmospheric Observatory of the Institute of Methodologies for Environmental Analysis of the Italian National Research Council (CNR-IMAA), CIAO (CNR-IMAA Atmospheric Observatory)^16^. The measurements were collected as part of the Mediterranean Experiment for Sea Salt and Dust Ice Nuclei (MESSA-DIN) in Soverato, South Italy (Latitude: 38.6894° N, Longitude: 16.545278° E, 30 m a.s.l.). One of the primary objectives of the campaign was to study aerosol-water vapor-cloud interactions, with particular emphasis on sea salt and dust. A ground-based remote sensing facility operated at the coastal site from June 24 to November 8, 2021. This paper focuses on water vapor measurements collected until September 30, 2021. During this period, frequent occurrences of high values of water vapor mixing ratio (WVMR) in the mid-troposphere were detected by the microwave radiometer, with retrievals constrained by an infrared thermometer and surface meteorological sensors. The retrieved profiles of WVMR were compared against the fifth-generation atmospheric reanalysis dataset, ERA5^17,18^. ERA5 cloudiness was also examined by comparing it with ceilometer and cloud radar observations, co-located with the microwave radiometer. Since ERA5 has been widely used for climate and modeling studies, comparisons with independent (i.e., non-assimilated) observations are essential for evaluating its performance during the extreme events. Elevated mid-tropospheric WVMR values observed during the study period were examined in relation to the prevailing synoptic configuration using ERA5 reanalysis data, to elucidate the combined role of synoptic and mesoscale water vapor transport in modulating the observed atmospheric conditions. To further investigate the origin and pathways of the moisture responsible for the observed mid-tropospheric enhancements, we additionally performed a Lagrangian trajectory analysis. This analysis also aimed to characterize the synoptic-scale circulation patterns governing moisture transport over the Mediterranean Basin during summer 2021, with particular attention to (i) the vertical layers sustaining long-range moisture advection and (ii) the relationship between enhanced tropospheric water vapor transport, fed by both remote and local moisture sources, and the occurrence of high-impact weather, such as the severe floods that affected Central and Eastern Europe in July 2021.

## Instruments and data

The study employs atmospheric observations from a suite of advanced instruments, including a microwave radiometer complemented by an infrared radiometer, a Ka-band Doppler radar, a laser ceilometer, a wind Doppler lidar, a UV polarization Raman lidar, a sun photometer, a total sky imager, and other near-surface measurements.

The Radiometrics Microwave Profiler (MWP-3014) provides retrievals of integrated water vapor (IWV), liquid water path (LWP), and temperature and humidity profiles^16,19^, using a neural network algorithm trained on coastal and mountain radiosonde observations^20^. Beyond occasional interruption, measurements were conducted continuously until a power supply failure occurred on 30 September. Depending on the retrieval type, the effective vertical resolution of temperature and WVMR profiles degrades approximately linearly with height, from a few hundred meters near the surface, to about 1 km at 1.5 km altitude, and up to around 5 km in the upper troposphere. Calibration was ensured via the tipping curve method^21^. The bias in temperature retrievals is ± 0.5 K in the boundary layer, while it exceeds ± 1 K above^22^. The water vapor mixing ratio retrieval typically exhibits biases on the order of ± 0.5 g kg^-1^ from the surface up to about 4 km, decreasing progressively at higher altitudes^23–27^. The standard deviation of the bias is largest within the boundary layer, reaching values up to ~ 1.5 g kg^-1^, while it decreases above and remains around ~ 0.5 g kg^-1^ in the free troposphere^24,25,27^.

The Vaisala CL51 ceilometer measured cloud base heights and attenuated backscatter coefficient profiles up to 15 km a.g.l., with high temporal (10 s) and vertical (10 m) resolutions using a 910 nm laser diode system, though limited by a low signal-to-noise ratio^28^. Data were processed using the ACTRIS-Cloudnet algorithm^29^, which provided the uncalibrated backscattering coefficient.

The Metek MIRA-36 Ka-band Doppler radar, designed for unattended long-term operation, provided range resolved cloud reflectivity, vertical wind, and linear depolarization ratio up to 15 km with 30 m vertical and 30 s temporal resolutions. The radar is also crucial in the detection of giant aerosol^16,30,32,33^.

Another key data source used in this study is the ERA5 reanalysis, which provides essential information for analyzing tropospheric water vapor transport and allows for the comparison of its data products with both in-situ and ground-based remote sensing observations. ERA5 is produced by ECMWF using a fixed version of Integrated Forecasting System (IFS), Cy41r2, and a four-dimensional variational (4D-Var) data assimilation system, with 137 hybrid model levels extended from the surface to 0.01 hPa at a 0.25° grid-space (highest spatial resolution available among global reanalyses). Higher spatial resolution by regional reanalyses (e.g., CERRA, UERRA) have not been covering the study period at the time of working; therefore, they are not included in this study, but their potential usage is discussed in “Conclusions” section. However, comparative performance analyses of CERRA and ERA5 (Ridal et al. 2024^31^) did not reveal notable improvements for water vapor, either at the surface or in the free troposphere, unlike improvements seen for temperature and wind fields. For ERA5 comparison with observational measurements, the hourly data is extracted for the nearest grid-point to the measurement site for the period from June to September 2021 at the tropospheric levels from 1000 hPa to 300 hPa. The choice of this single grid point is based on an evaluation of the 300–500 hPa layer over the Soverato site, where comparisons with multiple nearby grid points indicate that alternative selections have only a marginal impact on the results across the surrounding region (see Figure S6). Moreover, bilinear interpolation of ERA5 grid values showed differences with the nearest neighbor approach smaller than approximately ± 0.3 g kg-1 in mixing ratio across all pressure levels between 300 and 1000 hPa. In particular, in the range 450–650 hPa the bilinear interpolated minus nearest neighbor time series of the WVMR showed a difference generally smaller than 0.1 g kg-1.

To characterize the high WVMR values observed during the campaign in the mid- and upper troposphere, as well as their relationship with water vapor transport from remote or local sources, the ERA5 data have been used to estimate Integrated Vapor Transport (IVT), as the intensity of the IVT vector^34^:

$$\:{IVT}=-\frac{1}{g}\int\limits_{1000}^{300}{v}qdp$$

where g is the gravitational acceleration, v is the wind speed, q is the specific humidity, p is the pressure, and the integration is from 1000 to 300 hPa. Vectors are noted in bold. The period from June 24th to September 30th was characterized by high IVT values in different areas of the Mediterranean basin, involving also Soverato Gulf (two GIF animations with a sequence of plots every 2-hours, for the periods 1–5 July and 11–15 July, are provided in the supplementary materials).

To better characterize the identified moisture source regions, the evaporation of water equivalent, provided by ERA5, has been also analyzed, which quantifies the amount of water transferred from the surface to the atmosphere through evaporation and transpiration. The water vapor originated predominantly from zonal transport coming from the Atlantic Ocean, as well as from evaporation processes over the Tyrrhenian Sea and the Gulf of Lion. Evaporation sources were also identified over the Gulf of Soverato, as well as along the nearby northern African coast.

To better investigate water vapor transport, the IVT was analyzed also for two distinct pressure layers:

$$\:{{I}{V}{T}}_{300/700}=-\frac{1}{g}\int\limits_{700}^{300}{v}qdp$$

$$\:{{I}{V}{T}}_{700/1000}=-\frac{1}{g}\int\limits_{1000}^{700}{v}qdp$$

This vertical separation allows us to better assess the respective contributions of upper- and lower-level transport to the overall moisture advection, with particular focus on the region where the MP3014 instrument at the Soverato site recorded a significant increase in the WVMR.

Finally, the NOAA HYSPLIT trajectory model^35,36^ has been used to calculate 48-hour forward and 72-hour backward trajectories, initialized every hour from the Soverato site, at four altitude levels (3000, 4000, 5000, and 6000 m a.s.l.) in the mid-troposphere. Model vertical velocity was used in the simulations, which were driven by NCEP/NCAR reanalysis data. The water vapor mixing ratio along each trajectory was subsequently calculated from the corresponding meteorological variables.

## Results

The atmospheric circulation in summer 2021 was dominated by a strong ‘blocking high’ pressure system across southeastern Europe^37^. This system initially expanded towards the east and was followed by another high-pressure system further west^15^. These conditions favored dry weather and heatwaves with exceptionally high temperatures^14^, initially affecting the eastern and central Mediterranean areas and the Balkans, and then spreading to Spain, lasting until mid-August.

**Fig. 1:**
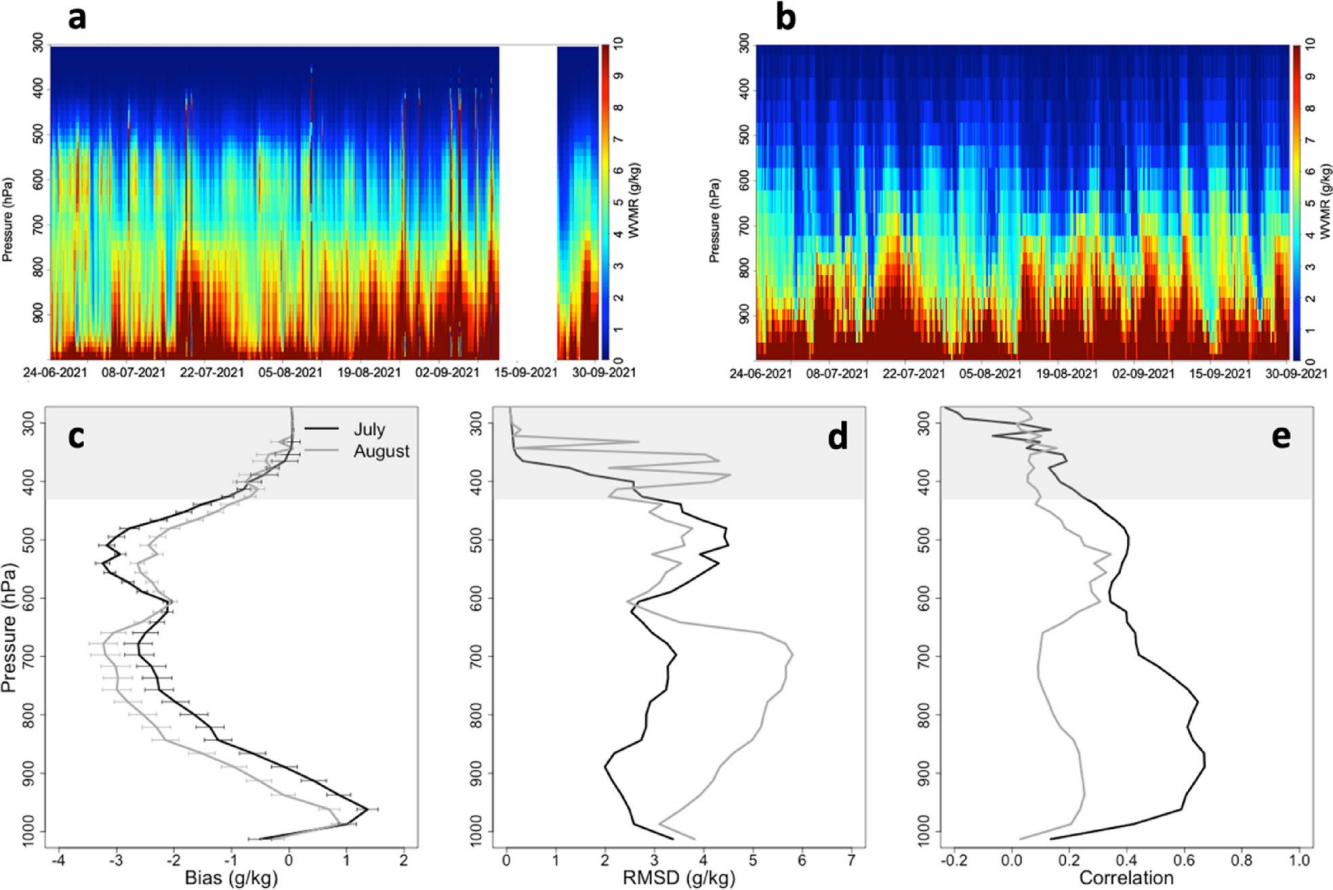
(**a**) Water vapor mixing ratio profiles from 300 to 1000 hPa, estimated using the microwave profiler (MWP) neural network retrieval from 24 June to 30 September 2021, with a time resolution of 5 min. The last altitude level is set at 10 km. (**b**) Same as panel (**a**) but obtained using ECMWF ERA5 hourly reanalysis data. Panels (**c**), (**d**), and (**e**) show the bias (ERA5 minus MWP), root-mean-square deviation (RMSD), and correlation between ERA5 and the MWP for July (black line) and August 2021 (grey line), obtained by averaging the MWP over 1-hour intervals at full hours. The standard error to the bias is also reported in the panel (**c**) (horizontal error bars). Finally, the gray shaded area at the top of the plot indicates the pressure range where the MWP retrievals approach a climatological mean.

During the measurement period from June 24th to September 30th, the MP3014 observed frequent and pronounced intermittent increases in water vapor content in the mid-troposphere, with high values of the water vapor mixing ratio (WVMR) in the altitude range between 450 and 650 hPa (Fig. 1, panel a). The time series shows intense moist structures, periodically disappearing, with values of WVMR of 5–6 g kg^-1^ and rare higher values. Intermediate periods were characterized by WVMR values below 4 g kg^-1^. Freezing level was positioned at 4–5 km a.g.l., as derived from the MWP temperature retrievals. The time series showed these high values on several days until August 10th, less frequently in the remainder of August and early September, and then again in late September. Low-pressure systems bringing clouds and rain, as well as a major issue to the measurements site power supply network, reduced and hindered reliable observations with the MWP during the full month of September. Very infrequent low clouds or short showers also affected a very minor fraction of the MWP observations (clearly visible in Fig. 1, as profiles saturating the color scale in a large vertical range).

It is worth noting that retrievals applied to microwave brightness temperatures at altitudes above 5 km, where the measurements remain sensitive to water vapor, are characterized by a coarse effective resolution^25,38^ and may be influenced by the climatological values related to the input training datasets. This may introduce significant errors in the retrieved values^20^. However, in terms of capturing the vertical gradient of humidity in the mid-troposphere, the neural network retrieval has already demonstrated to be quite efficient^16^. This is also due to the availability of the sky temperature measured with the infrared thermometer (IRT), used to constrain the retrieval, indicating the presence of high water vapor concentrations or cloud occurrence within the 9.6–11.5 μm spectral window region. During the campaign period, the IRT measured very frequent high temperature values of the clear sky, around 250–260 K (Figure S1). These temperatures are consistent with the presence of a large amount of water vapor in the middle troposphere.

In Fig. 1 (panel b), the time series of the WVMR provided by ERA5, which is extracted for the nearest grid point to the Soverato site (Figure S2), is also shown. The WVMR of ERA5 in the range 500–650 hPa is mainly up to 5 g kg^-1^ with very rare higher values, though decreasing above to values lower than 3 g kg^-1^, with a daily variability generally in good agreement with the MWP retrievals. In Fig. 1, the bias with its standard error (panel c), root-mean-square deviation (RMSD, panel d), and correlation (panel e) between ERA5 and the MWP are also reported for the month of July and August 2021, both covered by almost continuous measurements (short breaks in July, only). The bias is positive from the surface to 900 hPa only, with value up to 1 g kg^-1^, while above is always negative, with values up to −3 g kg-1 at 700 hPa and 500 hPa for the month of August and July, respectively. The standard error of the bias, quantifying the uncertainty in the ERA5-MWP difference, remains generally below 0.5 g kg^-1^ and decreases with altitude, indicating that the discrepancy between the two datasets becomes more stable and less variable in the upper troposphere. Above 500 hPa, the WVMR and the bias progressively reduces about to zero. The RMSD for the month July is almost constant until 600 hPa at a value of 3 g kg^-1^, while for August RMSD exceeds 4 g kg^-1^. In the range 450–650 hPa, the situation is the opposite with higher values in July (up to 4 g kg^-1^) than in August (about 3 g kg^-1^). Above, the RMSD progressively decreases. The correlation is higher in July than in August: in July, it reaches up to 0.6 from the surface to 700 hPa, while in the moist vertical region identified by the MWR retrievals it is around 0.4. In August, the correlation is lower, ranging from approximately 0.2 to 0.3 throughout the entire profile.

**Fig. 2:**
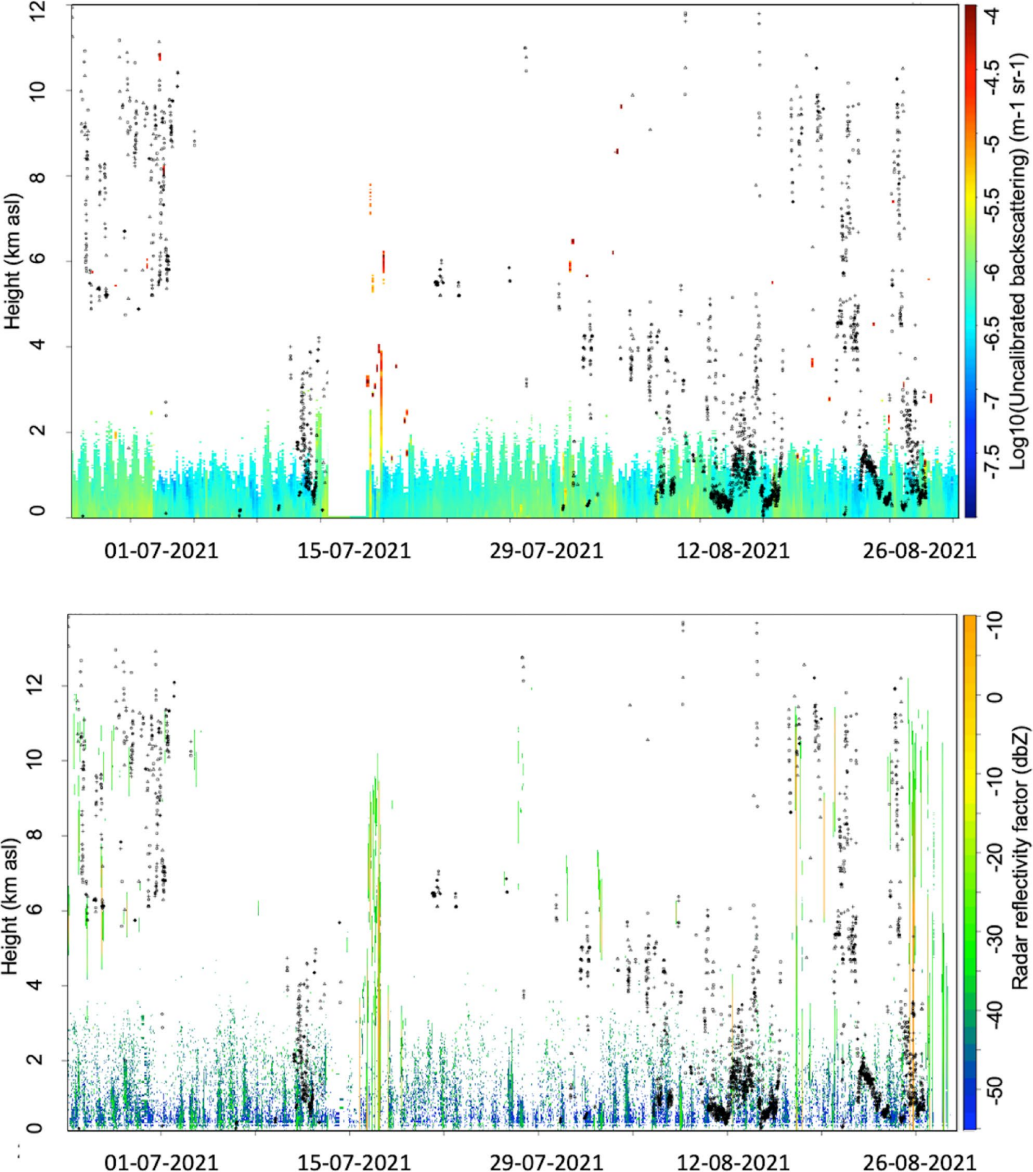
Top panel: uncalibrated aerosol backscattering coefficient (in dB) from the CL51 laser ceilometer, along with cloud base height estimates (black dots) derived from the ERA5 single-level dataset at the closest grid points to the Soverato location, in the period from June to August 2021; bottom panel: same as the top panel but showing equivalent radar reflectivity. The radar reflectivity factor, which is proportional to the sixth power of particle diameter, is used to detect clouds and precipitation. During the measurement campaign, values greater than − 30 dBZ were associated with hydrometeors, while values below − 40 dBZ were typically attributed to non-meteorological echoes, such as aerosols, pollen, or insects.

In terms of cloudiness, the time series of the uncalibrated backscattering (Fig. 2, top panel) and the radar reflectivity factor (Fig. 2, bottom panel), measured respectively with the laser ceilometer and the cloud radar operating at the site, are compared with the ERA5 cloud-base height from the four nearest grid points to the measurement location in the period from June to August 2021. Until 2 July, ERA5 systematically overestimated the presence of clouds in the 5–11 km a.s.l. layer. A relatively good agreement between ERA5 and cloud-radar observations was observed during the first half of the last week of June; however, in the second half of the same week, ERA5 continued to detect high clouds above 6 km that were not observed by the ground-based remote-sensing instruments.

A comparison of ERA5 cloud cover at Soverato with observations was carried out by estimating the same variable from the ground-based instruments using cloud-base height measurements provided by the Cloudnet classification scheme^29^.

Cloud base heights measured by the ceilometer were used to identify cloudy conditions, and the hourly cloud fraction was calculated as the proportion of clouds within ± 30 min of each nominal hour. Clouds were categorized as high-level or mid- to low-level based on mean cloud-base height. When ceilometer data were missing, ERA5 cloud-base heights were used as a fallback, yielding a hierarchical cloud-type classification.

These observational cloud fractions were compared with ERA5 cloud cover at matching times. A low threshold (0.01) was applied to define cloud presence for both observations and ERA5 data records, allowing thin or cirrus clouds to be included. Contingency analyses were performed within a conditional sampling framework, in which only time steps with detected cloud occurrence were retained. The analysis focuses on the Probability of Detection (POD), False Alarm Ratio (FAR), and Critical Success Index (CSI), which provide an appropriate assessment of cloud detection skill under conditional sampling.

For high clouds, ERA5 successfully detects 100 cloud occurrences, with 22 missed detections and 15 false alarms. The resulting POD of 0.82 indicates a high detection capability for high-level clouds. The FAR of 0.13 suggests a relatively limited overestimation of cold-cloud occurrence, while the CSI of 0.73 reflects good overall skill when considering both missed and falsely detected events.

For mid- and low clouds, ERA5 identifies 308 cloud occurrences but also produces 355 false alarms and 31 missed detections. The high POD of 0.91 demonstrates that ERA5 is generally able to capture the presence of low- and mid-level clouds. However, the large FAR of 0.54 reveals a pronounced tendency to overestimate warm-cloud occurrence. This behaviour is reflected in a lower CSI of 0.44, indicating reduced overall skill compared to high cloud detection.

A sensitivity test was conducted using a higher cloud-fraction threshold (CF > 0.20), consistent with the threshold adopted in numerical weather prediction (NWP) models for classifying overcast or predominantly cloudy conditions. Under this more conservative criterion, the ability of ERA5 to detect clouds was reduced, particularly for high-level clouds: ERA5 correctly identified 50 cloud occurrences, with 32 missed detections and 21 false alarms, yielding a POD of 0.61, FAR of 0.30, and CSI of 0.49. For mid- and low level clouds, the higher threshold resulted in 160 correctly detected clouds, 72 missed events, and 154 false alarms, corresponding to a POD of 0.69, FAR of 0.49, and CSI of 0.41. Additional tests using shorter temporal aggregation windows of ± 10 or ± 5 min around each nominal analysis hour showed that POD remained almost unchanged, while the FAR and CSI were slightly degraded, reflecting increased mismatches between Cloudnet observations and ERA5 reanalysis at finer temporal scales.

It is important to note that both the representativeness mismatch between the point-like Cloudnet observations and the grid-box–averaged ERA5 fields, as well as the temporal aggregation of high-frequency measurements into hourly windows, may introduce non-negligible sources of uncertainty.

**Fig. 3:**
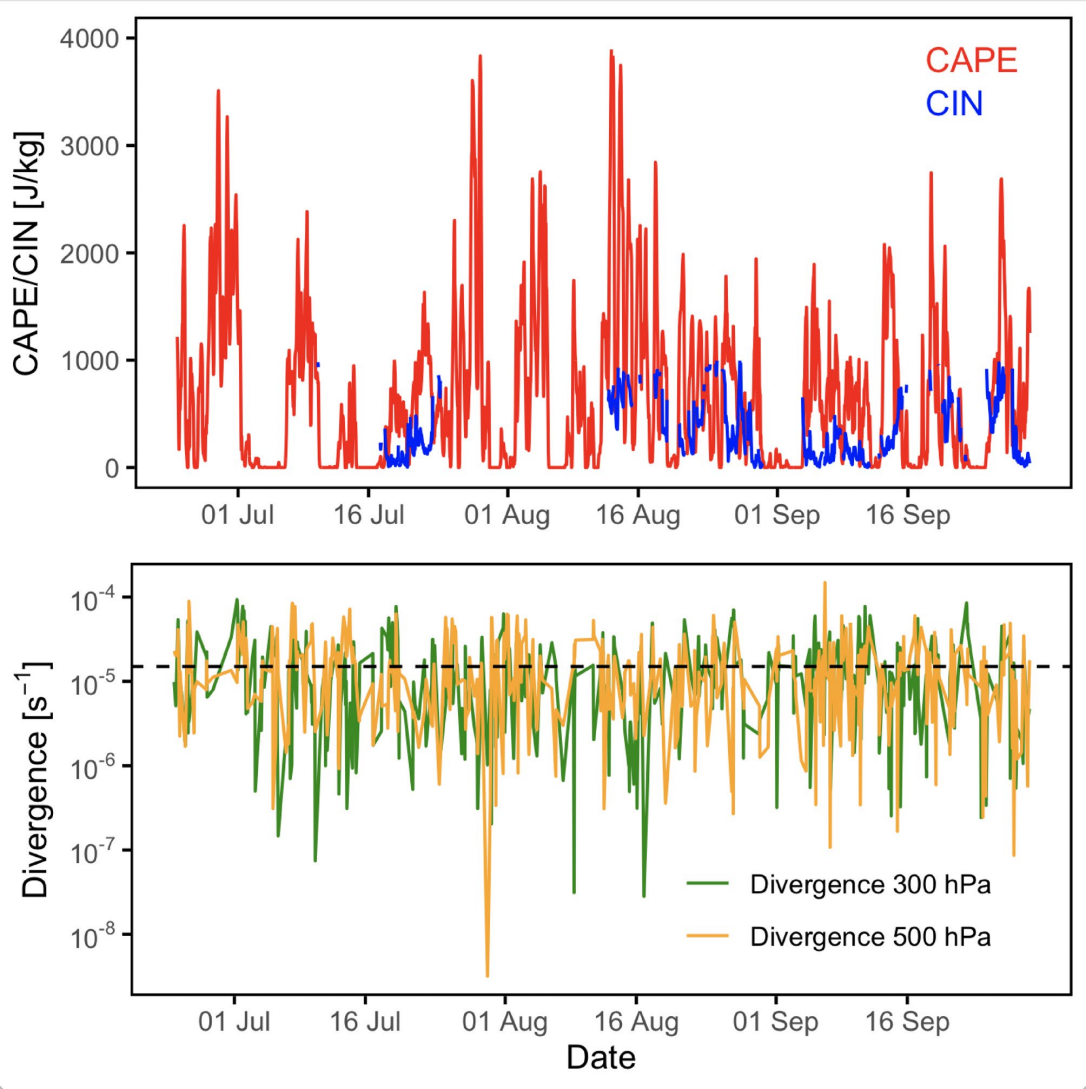
Top panel, Convective Available Potential Energy (CAPE) and Convective Inhibition (CIN) retrieved from ERA5 reanalysis data for the period 24 June to 30 September 2021; bottom panel, and divergence at 500 hPa and 300 hPa from ERA5 for the same period of top panel.

The occurrence of heatwaves during extended periods of the summer of 2021 likely inhibited strong convection and limited cloud formation. To clarify the purpose of the analysis, CAPE, CIN, and upper-level divergence at 300 and 500 hPa (derived from ERA5, Fig. 3) were examined as diagnostic indicators of the potential for convective activity under the observed synoptic conditions.

The CAPE index typically exceeds 1000 J kg^-1^ during episodes of moderate convection. Strong convection, which can trigger intense weather events such as sudden summer storms and gusty winds, is generally associated with values ranging between 2500 and 4000 J kg^-1^. Figure 3 (top panel) shows that CAPE values only occasionally exceed 2500 J kg^-1^. While this does not entirely rule out episodes of strong convection, such events were short-lived, and the prevailing conditions were moderately favorable rather than persistently strong. Moderate convection becomes more frequent from mid-July onward. In contrast, several periods show low CAPE values, indicating shallow or absent convection. For instance, at the beginning of the observational campaign, coinciding with the exceptional heatwave in Sicily where temperatures exceeded 48 °C^14^, convection was classified as moderate to strong. The CIN remained close to zero until the first half of August, except for a ten-day period in the second half of July and several days in September. Near-zero CIN in this period is physically consistent with a strongly heated Mediterranean boundary layer, where turbulent mixing and progressive erosion of the stable layer frequently eliminate most of the convective inhibition. In the second half of August and throughout much of September, CIN values frequently exceeded 200 J kg^-1^, indicating strong inhibition of convection during that time.

Upper-level divergence at 300–500 hPa (Fig. 3, bottom panel) serves as a dynamic indicator supporting vertical motion, even if CAPE remains moderate. Although it does not always coincide with the highest values of convective available potential energy (CAPE), it can nonetheless reflect synoptic-scale conditions generating sustained upward air movement. Although divergence values at 300 hPa occasionally exceed 0.5 × 10^− 5^ s^− 1^, reaching up to 2–3 × 10^− 5^ s^− 1^, they are not consistently strong enough to sustain deep convection in the absence of significant CAPE. Although CAPE and upper-level divergence indicate only moderate convective potential for most of the summer, CAPE and upper-level divergence remain the principal metrics for evaluating the local convective environment. Furthermore, as shown in Fig. 2 from the CL51 ceilometer measurements, the mixing layer height, identified by the vertical extent of aerosol mixing in the lower troposphere, remained consistently below 2.0 to 2.5 km above ground level (a.g.l.). This suggests that the region of most intense vertical mixing was relatively shallow, reducing the likelihood of deep convective development. It is worth adding that a regional climatology of the CAPE and CIN, spanning 1994–2020 (values are reported in the Table S1 of Supplementary information) and calculated for each summer month (June–September) shows that the values observed in Soverato were moderately elevated relative to the historical record. A peak in August stands out as an exceptional feature of the 2021 summer, reflecting increased convective potential and inhibition compared to typical years. However, all values remain within the 10th−90th percentile range, confirming that these deviations are not extreme.

In addition, using microwave radiometer and co-located wind Doppler lidar data, time series of equivalent potential temperature (θ_e_), temperature lapse rate, and vertical wind velocity have been investigated (Figure S3-S5). In regions of water vapor transport, mid-tropospheric θ_e_ values typically range between 330 and 350 K (Figure S3), consistent with warm and moist air masses. Up to approximately 950 hPa, the lapse rate (Figure S4) is generally smaller than the dry adiabatic lapse rate, indicating shallow temperature inversions, but it intermittently increases toward values near the dry adiabatic lapse rate. After August, convective activity increases as the lapse rate becomes higher below 900 hPa, promoting stronger vertical motion. Between 950 and 900 hPa, the lapse rate consistently reaches values up to 10 K km^-1^, indicating conditions close to dry-adiabatic extending also above. In the 450–650 hPa layer, the lapse rate decreases to 4–5 K km^-1^, reflecting moist-adiabatic conditions associated with water vapor transport. Finally, the wind vertical velocity (Figure S5), with most of the values within − 0.5 to 0.5 m s^−1^, does not reveal specific evidence of a correlation between vertical wind speed and the highest values of WVMR provided by the MWP at 450–650 hPa.

Despite occasional low clouds in July and August, the CL51 and cloud radar measurements (Fig. 2) in Soverato did unfrequently detect overcast sky conditions during summer, emphasizing the value of combination of MWP/IRT measurements in detecting large concentrations of water vapor in the free troposphere. In August, ERA5 frequently detected clouds below 5 km a.s.l., which were often not corresponding to the observations, indicating the challenges of ERA5 in properly representing convection and warm cloud formation in the measurement campaign area. However, it must be highlighted that measurements at the Soverato site were conducted at a coastal location with a rising orography leaving the coast in a South-East direction (Figure S2). This geographic and meteorological setting adds complexity to forecasting cloud formation of both synoptic and convective nature within models. This complexity is also reflected in ERA5 in a region where satellite measurements are the primary source of support for the reanalysis data, due to the absence of upper-air and ground-based remote sensing data. Moreover, it is worth noting that, despite the relatively high resolution of ERA5 compared to other global reanalyses, its spatial scale is still insufficient to explicitly resolve convection, which must therefore be parameterized. Additionally, it is well known that the IFS model exhibits inherent limitations in representing strong convective processes, particularly in coastal regions as documented in the ECMWF Forecast User Guide (Section 9.6.1) and highlighted in previous studies^39^.

**Fig. 4:**
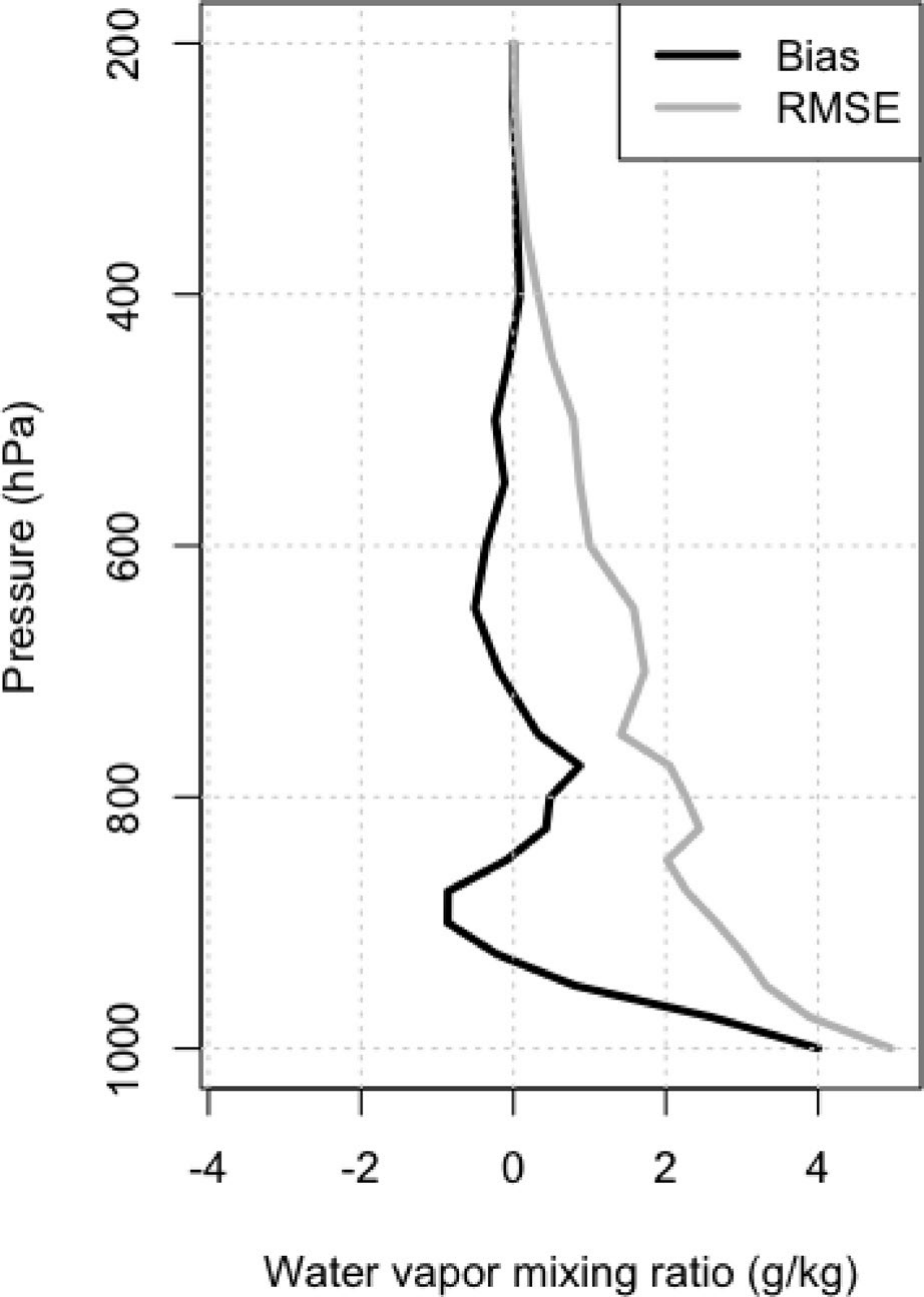
Bias and root-mean-square error for the ERA5 water vapour mixing ratio data (g kg^-1^) relative to upper-air measurements at the GRUAN station in Potenza, Italy (WIGOS ID: 0–20008-0-POT; 40.60° N, 15.72 E, 760 m asl), in the period June-September 2021.

An additional comparison to investigate the ERA5 dry bias in the campaign period is presented in Fig. 4, which shows the WVMR difference between ERA5 and measurements from upper-air sounding balloons launched at Potenza GRUAN (GCOS Reference Upper-Air Network) station (WIGOS ID: 0–20008-0-POT; 40.60° N, 15.72° E, 760 m asl). GRUAN is a reference network that provides traceable measurements with quantified uncertainties^40^. GRUAN data products are not assimilated in ERA5 and, therefore, they are an independent reference. Potenza is the only GRUAN station in Italy and is located in the southern part of the country. It sits in the Apennine mountains, representing a much drier environment than Soverato. During the campaign, the Potenza station performed one weekly launch, which is the minimum requirement for GRUAN. As a result, the comparison with ERA5 was based on a sample of 15 ascents. Figure 4 compares the ERA5 WVMR bias and root-mean-square error (RMSE) profiles relative to GRUAN at nearest grid point to Potenza. The bias ranges from 4 g kg^-1^ and − 1 g kg^-1^ from the near surface to the upper troposphere, and it generally decreases with the height. While the bias in the 650-400 hPa layer is lower than − 0.3 g kg^-1^ much smaller than that observed in Soverato, by contrast, the RMSE exceeds 1 g kg^-1^ in the same range. The large RMSE values reflect substantial differences in the profile-to-profile comparison, even in a mountainous environment, where ERA5 has difficulty capturing both the temporal and vertical variability of water vapor. Moreover, as discussed later in this section, air mass forward trajectories from Soverato do not overpass Potenza GRUAN site, which can be considered more as a control location. Similarly, in the supplementary material (Figure S7), relative humidity (RH) upper-air soundings from the Trapani Birgi RDS station (WIGOS ID: 0–20001-0–16429; 37.9142° N, 12.4914° E, 7 m asl) are shown alongside ERA5 hourly time series from the nearest reanalysis grid point. These soundings, performed twice daily (at 00 and 12 UTC) during the campaign period, provide further context. For Trapani station, the dry bias in ERA5 is generally smaller than that observed in Soverato, likely because ERA5 assimilates the regular radiosonde data from Trapani. However, in both radiosonde and ERA5 time series, peaks in the RH (which is the measured variable from the radiosondes) are observed in the 400–600 hPa range with values up to 90% RH. Trapani station is often along the tracks of water vapor transports in the Mediterranean for the considered period.

A comparison of precipitable water vapor (PWV) estimates from the MP3014 microwave radiometer, a co-located sun photometer, and ERA5 reanalysis reveals overall agreement in the temporal evolution throughout the campaign. Despite differences in spatial and temporal resolution, the three datasets capture similar variability, with the MP3014 and sun photometer often providing more consistent results during clear-sky conditions. Further details and a comprehensive analysis of these comparisons are provided in the Supplementary Information (Figure S8).

Two representative case studies are presented to illustrate distinct synoptic conditions observed during the campaign, highlighting different moisture transport pathways and source regions, as further detailed in the supplementary material where the full temporal evolution is provided through animated IVT and evaporation maps. The two case studies were selected because they correspond to periods of high WVMR values observed at the Soverato site by the MWP, allowing a direct interpretation of the local measurements in terms of large-scale moisture transport and source variability.

**Fig. 5:**
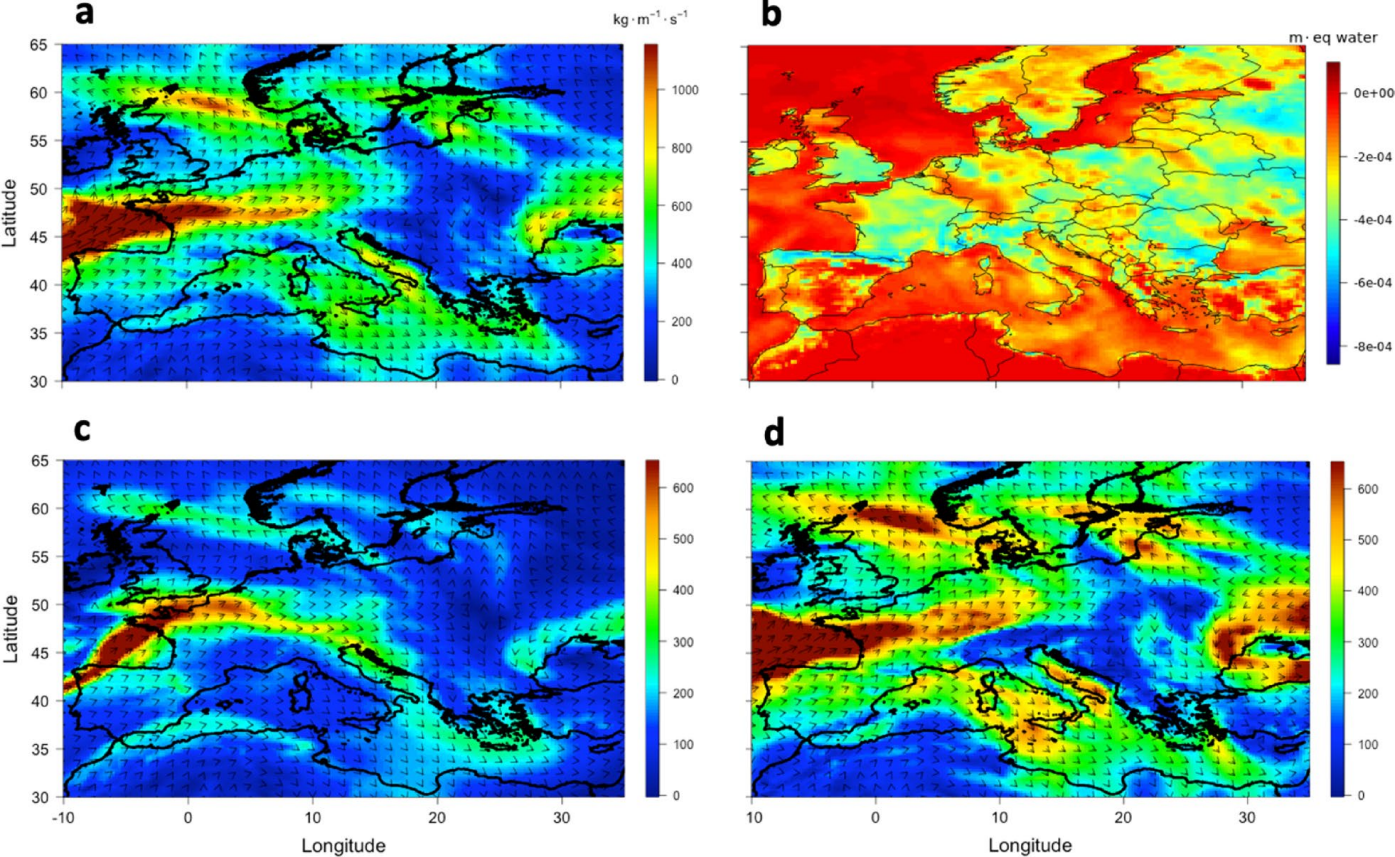
Map of the intensity and direction of the IVT vector and evaporation on 5 July 2021 at 12 UTC over Europe and the Mediterranean basin, obtained from ERA5. Panels a, c and d shows the IVT, $\:{IVT}_{300/700}$, and $\:{IVT}_{700/1000}$, respectively; panel b shows the hourly accumulated amount of water (in meters) that has evaporated from the Earth’s surface, including a simplified representation of transpiration (from vegetation), into vapor in the air above. Figure generated by the authors using custom scripts developed in R (version 4.3.x; https://www.r-project.org). The ERA5 reanalysis data is downloaded from are produced by the European Centre for Medium-Range Weather Forecasts (ECMWF) and made available via the Copernicus Climate Change Service (C3S). All data and figures are compliant with the CC BY 4.0 license.

Figure 5 shows the IVT for July 5th over the Mediterranean and Central Europe, highlighting a strong southeastward transport of water vapor, with values over the Soverato Gulf reaching 600 kg m⁻¹ s⁻¹, about half of which was transported within the 300–700 hPa layer. On this day, the water vapor transport was primarily zonal, originating from the Atlantic Ocean and presumably enhanced by secondary fluxes associated with intense evaporation over the Tyrrhenian Sea. This pattern is consistent with the direction of the mid- and upper-level winds observed in the synoptic setting, further supporting the key role of the 300–700 hPa layer in directing moisture toward the region of interest.

The zonal transport occurred along a deformation zone characterized by strong wind shear and the extension of the upper-level jet stream, which reached as far south as the Soverato Gulf, effectively channeling the moist air toward Southern Italy. On 5 July, Europe was positioned between two prominent anticyclonic ridges: a subtropical high extending northward from North Africa and a stable polar/Nordic high over North-East Europe (Figure S9). These upper-level features were aligned with corresponding surface pressure patterns. This configuration induced a pronounced meridional gradient in 500 hPa geopotential height, forcing the jet stream to traverse in the inter-ridge corridor^41–43^. The result was an enhanced and spatially focused upper-level jet streak, whose core traced a southeastward trajectory over the Mediterranean basin. As this jet passed above the warm surface waters of the North Atlantic and the Mediterranean, especially along the Gulf Stream and Tyrrhenian outflows, it encountered and entrained substantial moisture, which it subsequently advected towards Southern Italy. Quantitatively, nearly 50% of the total IVT over Soverato on that day was confined to the 300–700 hPa layer. This synoptic configuration is consistent with established theories on anticyclonic blocking patterns, where dual-ridge structures create a narrow meridional corridor that intensifies wind shear near the ridge exits and supports downstream jet acceleration^43^. In this case, the upper-level jet stream acted as the main transport mechanism for moist Atlantic air, guiding it through the geopotential corridor and delivering it into the mid-troposphere over Southern Italy. The resulting increase in lower-tropospheric moisture may generally contribute to enhanced convective instability in the region.

**Fig. 6:**
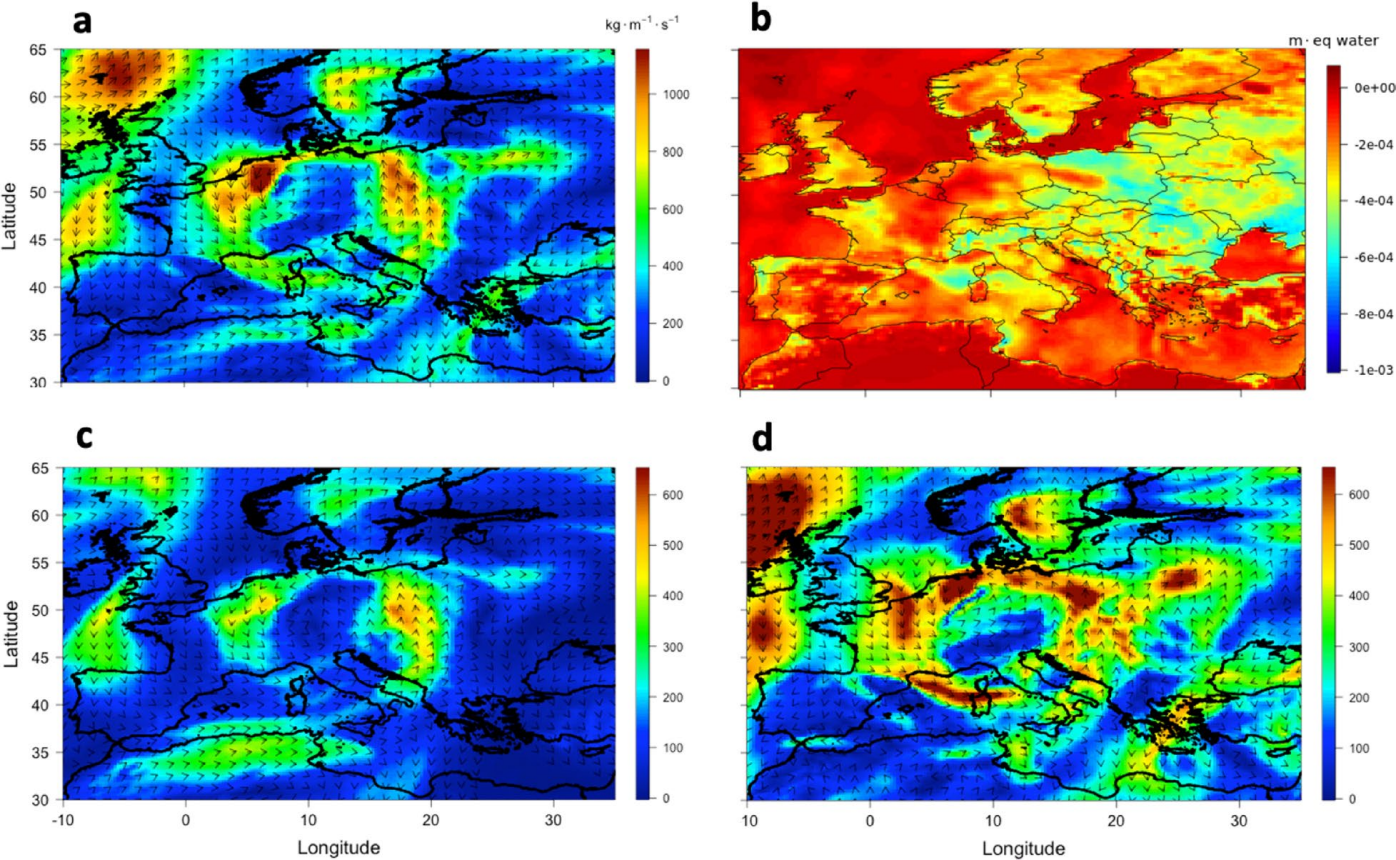
Same as Fig. 5, but for the 14 July 2021 at 12 UTC.

Another illustrative case is provided in Fig. 6, for 14 July, where the dominant moisture advection is supplied not solely from Atlantic zonal flows but crucially via a pronounced flux originating over North Africa. The water vapor was also entrained along streamlines that traverse the interstice between the North African ridge and an adjacent mid-latitude low-pressure system, before being drawn into the low-pressure envelope and then descending over Soverato (Figure S10). Simultaneously, the evaporation intensification over the Gulf of Lion, further enriches the lower troposphere with moisture^44–46^. The combined advection thus exhibits IVT values of approximately 500 kg m^−1^ s^−1^, with roughly half the transport occurring in the 300–700 hPa layer, reinforcing the notion of vertically structured moisture delivery through mid-tropospheric conveyor dynamics. Concerning the role of Gulf of Lion evaporation, this phenomenon can be attributed to strong latent heat fluxes (> 1000 W m⁻²) induced by cold northerly outflows, which destabilize the marine boundary layer and foster enhanced moisture uptake^47^. Such processes have been shown to supply latent heat and moisture efficiently into downstream cyclogenesis and convective systems.

The animated GIF, available in the supplementary material, covering the periods from July 1st to 5th and from July 11th to 15th, illustrates the spatiotemporal evolution of water vapor advection and evaporation fields, highlighting how the moisture sources discussed in this section contribute to the observed transport patterns. It visually reinforces the role of both the Atlantic inflow and Mediterranean evaporation, particularly from the Tyrrhenian Sea and the Gulf of Lion, in sustaining the enhanced moisture flux toward southern Italy. The analysis of water vapor transport is based on ERA5 reanalysis data, which, despite the differences with the MWP WVMR discussed above, offers strong performance in capturing synoptic-scale features with good temporal and spatial consistency.

The trans-regional moisture interplay also motivated the investigation of a potential correlation between the WVMR retrieval in Soverato with the extreme precipitation event occurred across Western Germany in mid-July 2021. Atmospheric tracking techniques and reanalysis-based moisture source attribution, applied especially by Sodemann and Zubler^48^ and corroborated in more recent atmospheric-river studies, indicate that both the North Atlantic and the Mediterranean are significant contributors of moisture to central European precipitation extremes^49^. During the 12–15 July episode, the synoptic configuration featured a quasi-stationary cut-off low pressure system that sustained a warm, moisture-laden flow on its northwest flank, drawing substantial vapor from these water bodies^50^. Complementary investigation via WAM2layers/FLEXPART and other Lagrangian methods confirmed that Mediterranean evaporation can account for a notable, if secondary, share of total moisture influx into Central Europe during such events^51^. Thus, the elevated IVT and WVMR peaks observed at Soverato may represent a measurement of the upstream reservoir feeding into the same moisture conveyor belt that intensified convective precipitation and triggered the German floods. This inference not only strengthens the physical plausibility of a coherent moisture trajectory from the Mediterranean through Southern Italy toward Central Europe, but also aligns with emerging evidence that regional hydrometeorological extremes often result from multi-basin moisture sources^52^. It is worth noting that comparisons between ERA5 and observations of the German flooding event indicated reliable pattern reproduction, although the magnitude of the event was underestimated^53^.

**Fig. 7:**
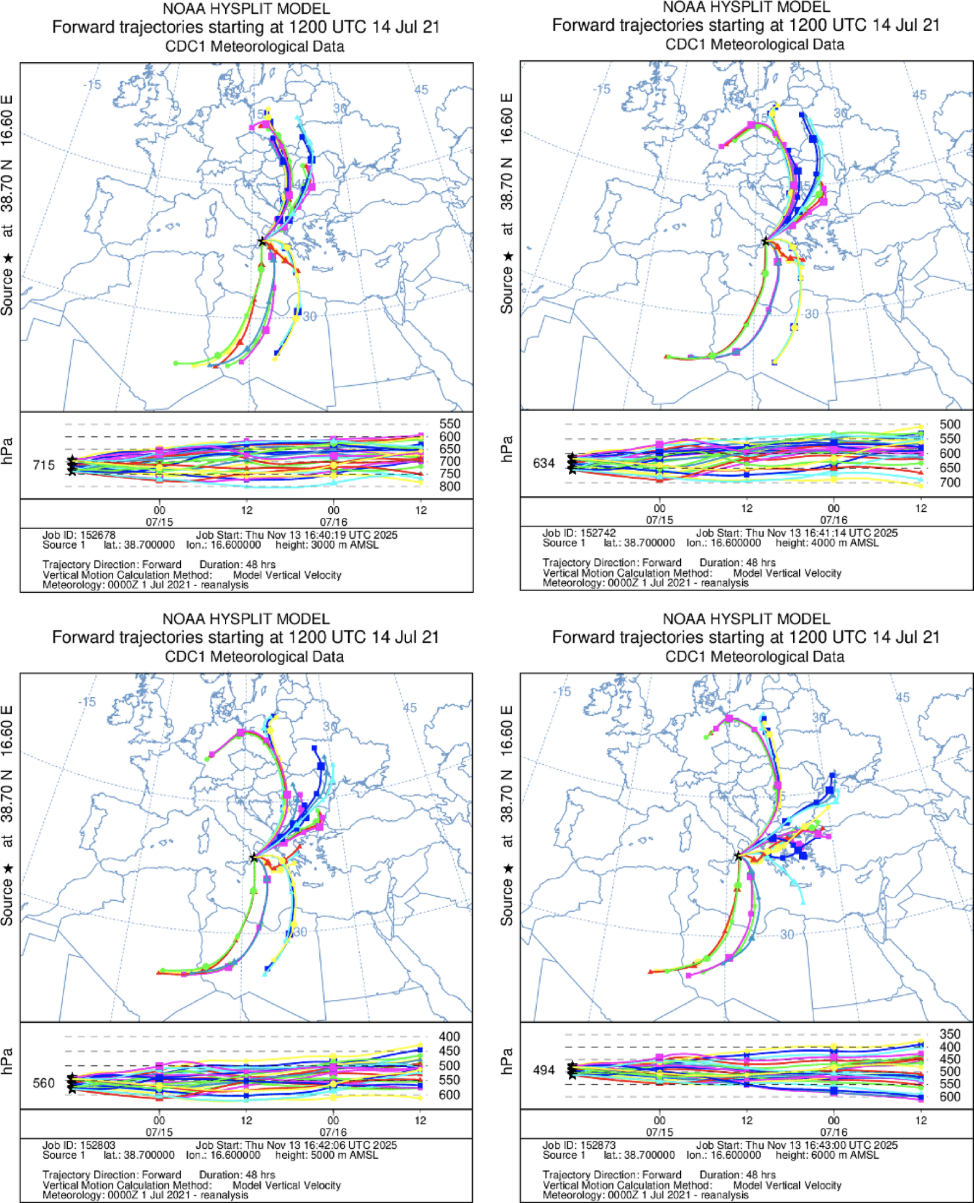
48-hour forward HYSPLIT trajectories initialized hourly from the Soverato site at four altitude levels (3000, 4000, 5000, and 6000 m a.s.l.). Trajectories are driven by NCEP/NCAR reanalysis data. Colors indicate the mixing ratio (g kg^-1^) along each trajectory. The figure shows the subsequent evolution of the moist air masses contributing to the synoptic situation and partly associated with the German flood event of mid-July 2021.

Therefore, the hypothesis of trans-regional water vapor transport observed on 14 July as a contributor to the enhancement of the low-pressure systems associated with the German flood event was further analyzed using both forward (Fig. 7) and backward (Figure S11-S12) HYSPLIT air masses trajectories: backward trajectories to identify the principal moisture source regions, and forward trajectories to characterize the subsequent air-mass evolution toward Central Europe, which is particularly relevant considering the concurrent synoptic conditions that generated floods over Germany. For 5 July 2021, only backward trajectories were computed to diagnose the dominant moisture sources (Figure S12). Although the trajectories are driven by the relatively coarse NCEP/NCAR reanalysis, this resolution is fully adequate for synoptic-scale applications, where the aim is to identify broad transport pathways and provide robust constraints on the relative contributions of major regional moisture-source regions, rather than to resolve fine-scale parcel motions. Lagrangian models such as HYSPLIT are routinely employed with global reanalyses to depict large-scale, dynamically consistent flow features^36^. The results are consistent with the analysis presented in Figs. 5 and 6, revealing a dominant role of Mediterranean and North Atlantic source regions in the observed water vapor transport.

On 14 July, Fig. 7 shows that several forward trajectories at altitudes above 600 hPa, after following the northwest flank of the low-pressure system, subsequently deviate toward central-western Germany, in qualitative agreement with the synoptic-scale conditions affecting the regions impacted by the floods. It must be stressed that such trajectory-based connections do not constitute a causal attribution, but rather illustrate a physically plausible transport pathway consistent with independent synoptic analyses.

72-hour backward trajectories from in Soverato mainly originate from northern Africa and they are associated with substantial moisture transport (Figure S13), with a WVMR variability showing values larger than 6 g kg^-1^ in the trajectory origin (within one standard deviation, though the trajectory ensemble reports values up to ~ 12 g kg^-1^). The Atlantic contribution was small at the lowest altitude but increased at mid- and high altitudes (25% at 4000 m, 40% at 5000 m, and 36% at 6000 m). Meanwhile, the fraction of moisture from the North Africa-Iberian Peninsula was highest at 3000 m (> 95%) and smaller at higher altitudes (60–75%).

The number of trajectories from Atlantic Ocean and Tyrrhenian Sea are the majority on 5 July, with WVMR variability showing values smaller than 5 g kg^-1^ (within one standard deviation, Figure S14). However, the fraction of total moisture originating from the Atlantic was relatively moderate at lower altitudes (18% at 3000 m and 17% at 4000 m), increasing at higher altitudes (20% at 5000 m and 23% at 6000 m). In contrast, the fraction of moisture coming through the North Africa-Iberian Peninsula corridor was substantially higher, ranging from 41% to 50%, indicating that this pathway strongly influenced air masses reaching the measurement site.

Finally, it is also worth noting that, despite the high WVMR values in the mid troposphere and the presence of diverse aerosol types, identified using UV polarization Raman lidar measurements, including marine, continental, and dust-contaminated particles, cloud occurrence at the Soverato site remained very limited during the campaign. This was primarily driven by persistent high-pressure conditions and temperature inversions that inhibited vertical air motion, thereby constraining both warm and high cloud formation, while the elevated mid-tropospheric moisture may have amplified greenhouse feedback^54^. A detailed overview of aerosol properties and variability is discussed in the supplementary material (Figure S15 and Table S2).

## Conclusions

The MESSA-DIN campaign, conducted at the coastal site of Soverato in southern Italy during the Mediterranean summer and early autumn of 2021, provided valuable insights into tropospheric water vapor transport and its contributions attributable to synoptic and mesoscale processes that modulated the observed atmospheric conditions. Observations revealed persistently high value of water vapor mixing ratio in the mid-troposphere throughout the campaign, driven by regional transport from both local and remote sources.

A microwave profiler and other instruments were used synergistically to provide a comprehensive understanding of water vapor profiles and cloudiness. The MP3014 time series of water vapor mixing ratio profiles were compared to corresponding data from the ERA5 atmospheric reanalysis. While ERA5 provided a coherent and detailed representation of synoptic patterns and showed general agreement in the temporal evolution of the atmospheric vertical structure with observations, it exhibited a dry bias in the water vapor mixing ratio up to 3.0 g kg-1 compared to the MWP in the 450–650 hPa vertical range. An uncertainty range of ~ 0.5 g kg^-1^ in MWP must be taken into account when quantifying the bias in the same pressure range.

In NWP models, low accuracy in representing water vapor transport affects the simulation of cloud formation. Our comparison between ERA5 data and local observations at Soverato indicates that, at a 0.25° resolution, ERA5 did not capture the short-term variability in water vapor at the Soverato site during the summer of 2021, under stable and aerosol-rich conditions. This may also be related to the fact that IFS versions released prior to summer 2022, such as the one used in ERA5, were affected by a non-conserving atmospheric water budget^55,56^, which can potentially impact the representation of water vapor.

The analysis also presented two representative case studies, associated with distinct synoptic conditions, which emphasize the critical role of both synoptic and mesoscale moisture transport in shaping local atmospheric conditions at the Soverato site and, more broadly, across the Mediterranean basin. Moisture originates from multiple sources, including the Atlantic and North Africa, with transport playing a particularly important role in enhancing humidity in the mid-troposphere at Soverato site (450–650 hPa).

The fractional moisture attribution reinforces this picture: the Atlantic contribution on 14 July was negligible at low altitudes but increased to 25–40% above 4000 m, while the North Africa-Iberian Peninsula sector dominated the moisture supply (60–100%) across all levels. A similar pattern emerged on 5 July, when despite a larger number of Atlantic and Tyrrhenian trajectories, the fractional moisture contribution from the North Africa–Iberian Peninsula corridor remained higher (41–50%) than that of the Atlantic (17–23%).

Furthermore, the observed IVT patterns and the HYSPLIT air-mass trajectories initialized from the Soverato profiles on 14 July show several forward trajectories initialized above 600 hPa subsequently curved toward Central and Western Europe along the northwest flank of the low-pressure system, in spatial and temporal agreement with air-mass pathways involved in the severe floods that impacted Western Germany in mid-July 2021. While this does not constitute a deterministic causal attribution, also considering the uncertainties of Lagrangian models, it provides coherent evidence supporting the plausibility of a broader moisture-transport connection between the Mediterranean and the regions impacted by the event.

Previous studies showed good performances of the IFS scheme and the associated data assimilation in predicting the temperature field through comparison with radiosonde and satellite observations^57,58^ and an improved regression fit for temperature, wind and humidity in the troposphere in comparison with radiosonde data prior to assimilation^18^. However, specific issues have been identified in the prediction and reanalysis of water vapour content in the upper troposphere and lower stratosphere, as well as with the general representation of ice supersaturation^58,59^. In the upper troposphere ERA5 is found to underestimate water vapor concentration and ice supersaturation^60,61^. Moreover, in comparison with homogenized datasets over the last four decades, also significant differences between radiosonde and reanalysis data have been found in the mid-upper troposphere, depending on the latitude of the comparison^62^. ERA5 dry-bias has been addressed either using multiplication factors or parameterized corrections, although proposed adjustments do not consider spatial variations in the bias, particularly at different pressure levels^63^. It must be pointed out that the assessment of the model performance can vary with time due to system versioning and potential changes in the data assimilation or model schemes with the new releases. For instance, IFS used during the forecasting time of summer 2021 (Cy47r2, implemented in May 2021) is different from the one implemented of 2016 to ERA5 (Cy41r2), and the present version (Cy48r1), which includes improvements to the representation of moist physics in the model and increased satellite observation usage in cloudy regions in data assimilation, in a few months after the highlighted flood event (in Cy47r3 release, implemented in October 2021).

For the analysis presented in this study, the dry bias of up to 3 g kg^-1^ in ERA5 relative to the MWP measurements in the mid-troposphere appears primarily attributable to limitations in the data assimilation system, which does likely not fully capture the contributions from both remote and local water vapor sources. Microphysical parameterizations and associated moisture biases likely contribute to discrepancies in cloud representation, affecting convective activity and radiative processes.

For cold clouds, defined using a hierarchical classification based primarily on Cloudnet retrievals of cloud-base height and supplemented by ERA5 when observational data were unavailable, ERA5 successfully detects 100 cloud occurrences, with 22 missed detections and 15 false alarms. The resulting POD of 0.82 indicates a high detection capability for high-level clouds. The FAR of 0.13 suggests a relatively limited overestimation of high cloud occurrence, while the CSI of 0.73 reflects good overall skill when considering both missed and falsely detected events.

For warm clouds, ERA5 identifies 308 cloud occurrences but also produces 355 false alarms and 31 missed detections. The high POD of 0.91 demonstrates that ERA5 is generally able to capture the presence of low- and mid-level clouds. However, the large FAR of 0.54 reveals a pronounced tendency to overestimate warm-cloud occurrence. This behaviour is reflected in a lower CSI of 0.44, indicating reduced overall skill compared to high cloud detection.

These results illustrate the challenges of accurately representing clouds at different altitudes, while uncertainties arising from the comparison of point-like Cloudnet observations and grid-box–averaged ERA5 fields may contribute additional variability in the evaluation.

The campaign results also emphasize the critical need for dense, high-quality water vapor measurements in regions like the Mediterranean, where complex transport from local and remote sources occurs. Discrepancies in water vapor and the lack of operational ground-based lidar or microwave radiometer networks highlight the importance of fully exploiting GRUAN data, targeted campaigns, and new observations, such as advanced radiosondes, GNSS Zenith Total Delay, and ceilometers, to improve reanalyses and forecast extreme events, potentially augmented through machine learning or statistical approaches. Experiments with indirect assimilation of microwave-derived temperature and humidity profiles have improved forecasts, although significant uncertainties remain^24^.

In conclusion, the findings of this study underscore the importance of accurately representing water vapor transport in weather and climate modeling, particularly in regions with complex structures, such as areas with pronounced orography or coastal environments. Intensified moisture advection, as highlighted by our results, may serve as a key precursor to high-impact precipitation events under specific synoptic conditions. Frequent and prolonged water vapor transport from different source areas can also affect the radiative balance, enhancing surface radiation trapping and potentially amplifying the impacts of heatwaves on humans^64^.

Although this analysis is based on a single measurement campaign at one site, and thus is constrained by the spatial and temporal coverage of available observations, the observed discrepancies with ERA5 water vapour mixing ratio and cloud data are consistent with known regional limitations of the reanalysis (e.g^62^.,, Ridal et al. 2024^31^). Further research should aim to extend the analysis to additional case studies, contingent on observational availability, and complement the results with numerical experiments.

## Supplementary Information

Below is the link to the electronic supplementary material.

Supplementary Material 1

## Data Availability

Cloudnet data is licensed under a Creative Commons Attribution 4.0 international licence. AERONET Data is available in near real-time on the AERONET website (https://aeronet.gsfc.nasa.gov/cgi-bin/data_display_aod_v3?site=Soverato_IMAA&nachal=2&level=2&place_code=10).The reanalysis data, ERA5, have been downloaded the Climate Date Store (CDS) of Copernicus Climate Change Service (C3S) which is publicly available through https://cds.climate.copernicus.eu/home.
