# Supplementary material for: Water vapor transport observed at a coastal Mediterranean site during the summer of 2021 and compared with ERA5
Source: Sci Rep. 2026 Feb 14;16:9105. doi: 10.1038/s41598-026-36040-0 (PMC12996470; doi:10.1038/s41598-026-36040-0)
Supplement: Supplementary file 1 — Supplementary Material 1 [file 41598_2026_36040_MOESM1_ESM.zip › Supplement_material/Supplement_material_v17122025.pdf]

Supplementary information to the manuscript "Water Vapor Transport observed at a Coastal Mediterranean Site during the Summer 2021 and comparison with ERA5", by Fabio Madonna et al.

This supplementary material provides additional figures and explanatory notes that complement and expand upon the analyses presented in the main manuscript. The included visualizations offer further detail on the considered datasets, methodological steps, and intermediate results that support the study's conclusions. These supplementary elements are intended to enhance the clarity and reproducibility of the work by documenting aspects that, for reasons of conciseness, could not be fully addressed in the main text.

Figure S1 shows the sky temperature measured with the infrared thermometer, used to constrain the retrievals of the microwave radiometer, indicating the presence of high water vapour concentrations or clouds within the spectral window region.

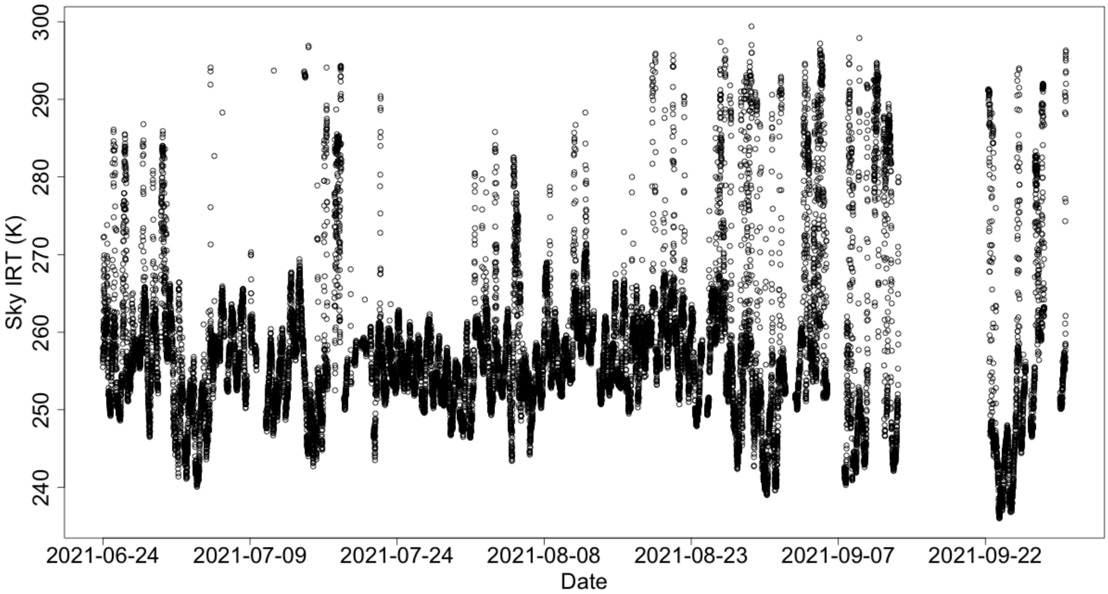

Figure S1: Infrared sky's temperature in the range of 9.6 to 11 microns as measured with the infrared thermometer, operating in synergy (same time sampling of 5 minutes) with microwave radiometer in Soverato.

Figure S2 shows an aerial map of the Soverato with an indication of the measurement site where the MESSA-DIN campaign took place.

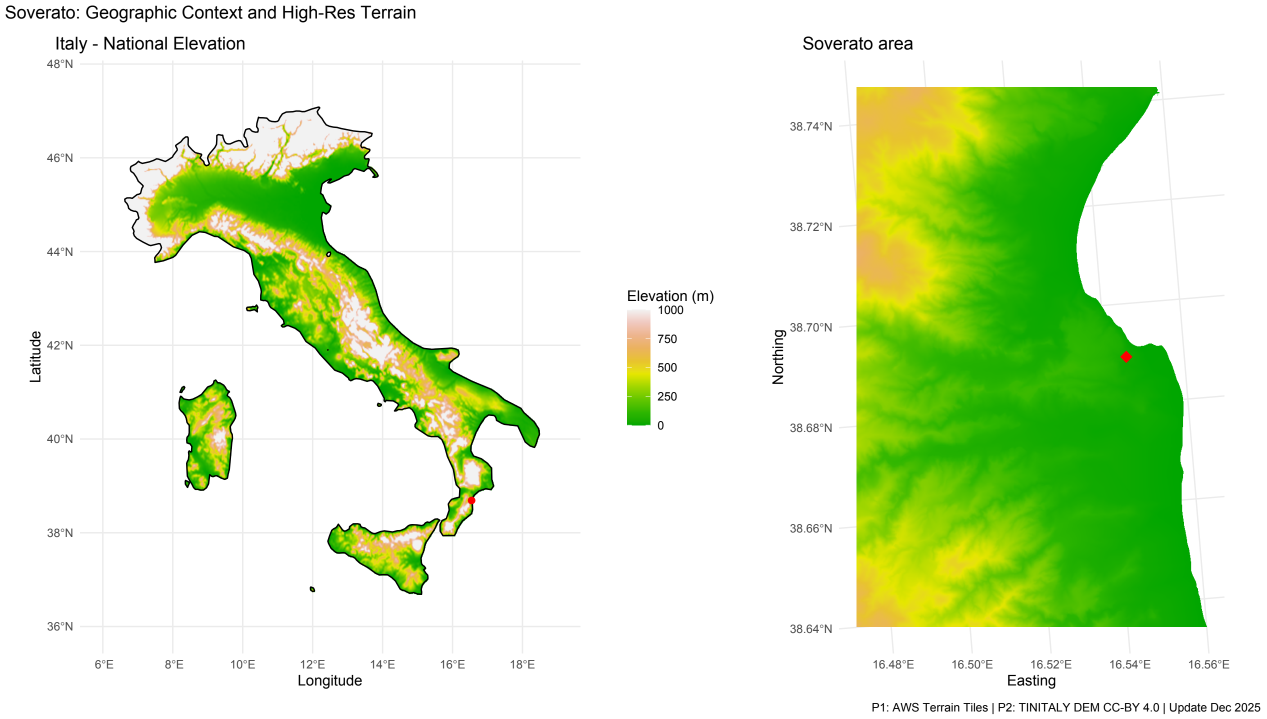

Figure S2: Digital Elevation Model (DEM) and geographic location of Soverato (Italy). The left panel (a) shows the national topographic context of Italy using terrain tiles provided by Amazon Web Services (AWS) via the elevatr package. The right panel (b) displays a high-resolution detail of the Soverato area based on the TINITALY DEM (v1.1) provided by the National Institute of Geophysics and Volcanology (INGV), released under a Creative Commons Attribution 4.0 International (CC BY 4.0) license [Tarquini et al. 2023, doi:10.13127/tinitaly/1.1]. National boundaries are derived from Natural Earth (public domain). The elevation color scale is capped at 1000 m to emphasize coastal and hilly morphological features. Maps were generated using R Statistical Software (v4.4.1) [www.r-project.org] and the following packages: terra (v1.8-21), sf (v1.0-19), ggplot2 (v3.5.1), elevatr (v0.9.9), and patchwork (v1.3.0).

Using microwave radiometer and co-located wind Doppler lidar data, we provide time series of equivalent potential temperature (θ_e), lapse rate, and vertical wind velocity (Figures S3-S5). In regions associated with water vapor transport, θ_e values retrieved in the mid-troposphere typically range between 330 and 350 K, consistent with warm and moist air masses. Regarding the lapse rate, in the layer extending up to approximately 950 hPa, it is predominantly negative, indicating frequent temperature inversions associated with shallow stratification and suppressed vertical mixing. Within this layer, however, the lapse rate intermittently alternates between negative and positive values. After August, convective activity intensifies, primarily because the lapse rate becomes predominantly positive below 900 hPa, thereby promoting enhanced vertical motion in the lower troposphere. Between 950 and 900 hPa, the lapse rate transitions to consistently positive values and subsequently increases with height. Above this level, the thermal stratification steepens progressively, reflecting a transition toward dry-adiabatic conditions and enhanced vertical temperature

gradients. In the 450-650 hPa range, the lapse rate decreases to approximately 4-5 K/km, indicative of moist-adiabatic conditions consistent with active water vapor transport. Finally, the wind vertical velocity, with most of the values within -0.5 to 0.5 m s^{-1}, does not reveal specific evidence of a correlation between vertical wind speed and the high values of WVMR provided by the MWP at 450-650 hPa.

Together, the θ_e and lapse rate fields provide a detailed and coherent depiction of both the mean tropospheric energy and moisture content and the active regions of vertical water vapor transport, complementing the time series already shown in the manuscript.

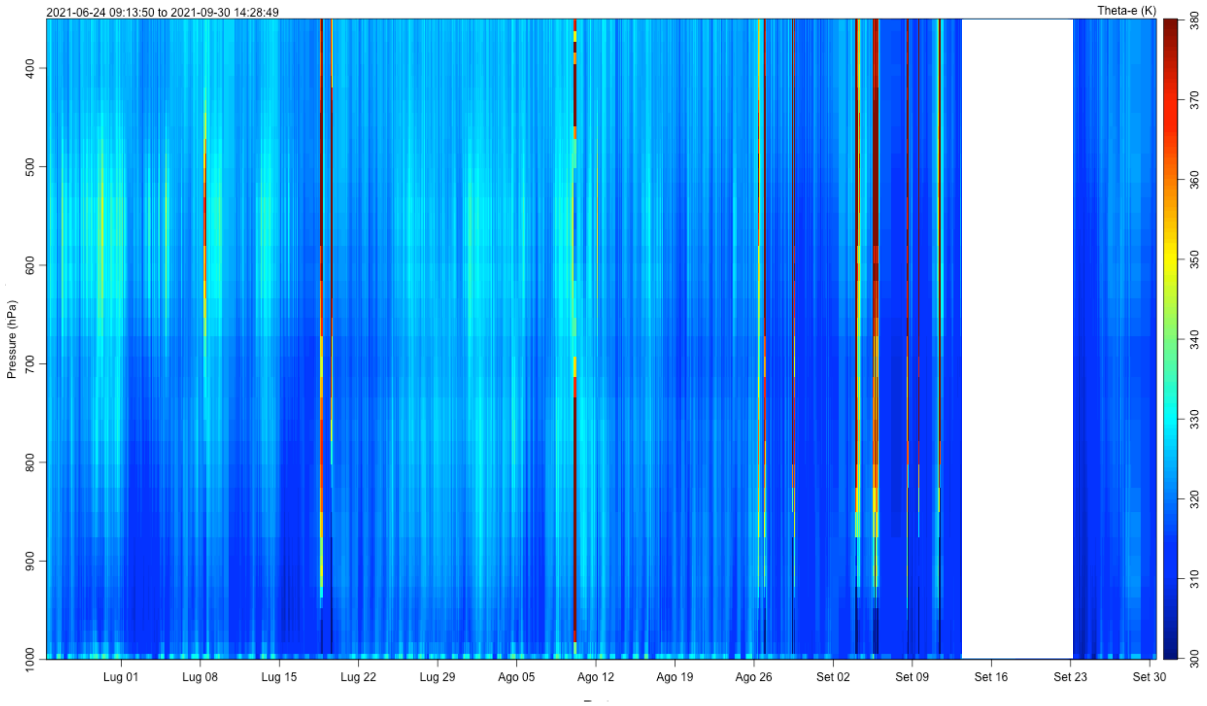

Figure S3: Vertical cross-sections of equivalent potential temperature (θ_e) retrieved from the microwave radiometer over the full observational period. Mid-tropospheric θ_e values (330-350 K) indicate the presence of warm and moist air masses associated with water vapor transport.

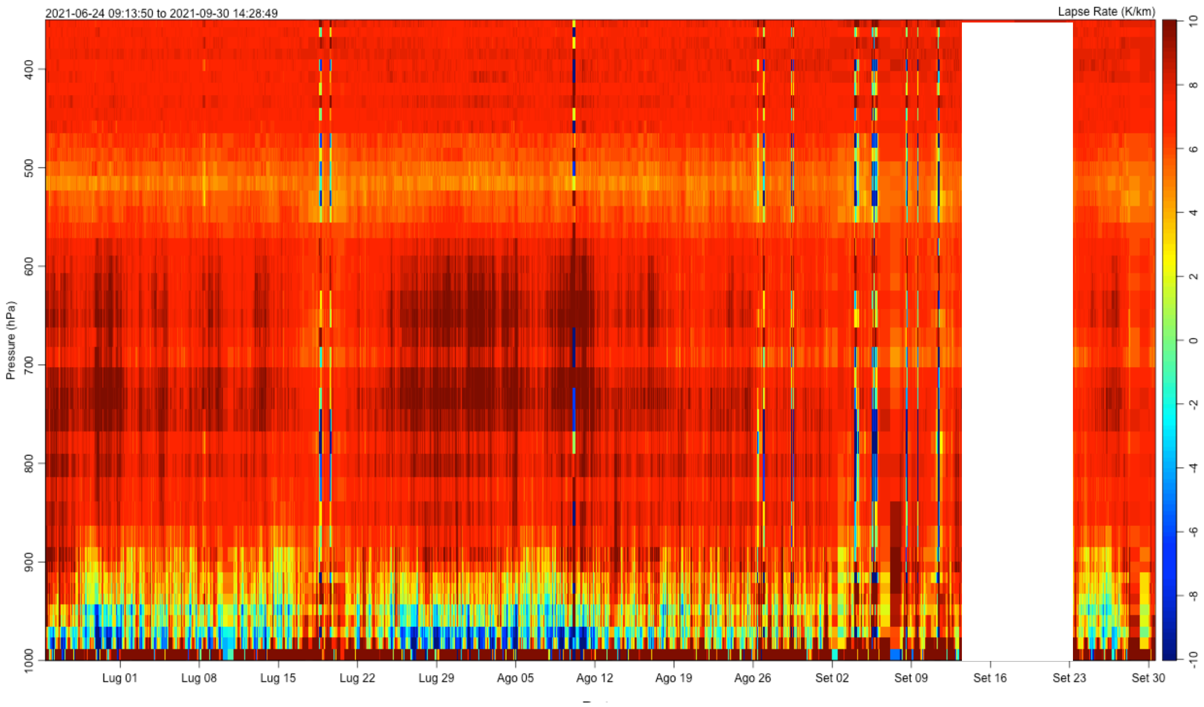

Figure S4: Lapse-rate fields derived from microwave-radiometer temperature retrievals. Negative values in the lower troposphere indicate convective boundary-layer conditions, while strongly positive lapse rates (about 10 K km^{-1}) between 600-700 hPa reveal a highly stable layer during June-July and early August. Intermediate values (4-5 K km^{-1}) in the 450-600 hPa layer mark the transition toward less stable regions in which vertical water vapor transport preferentially occurs.

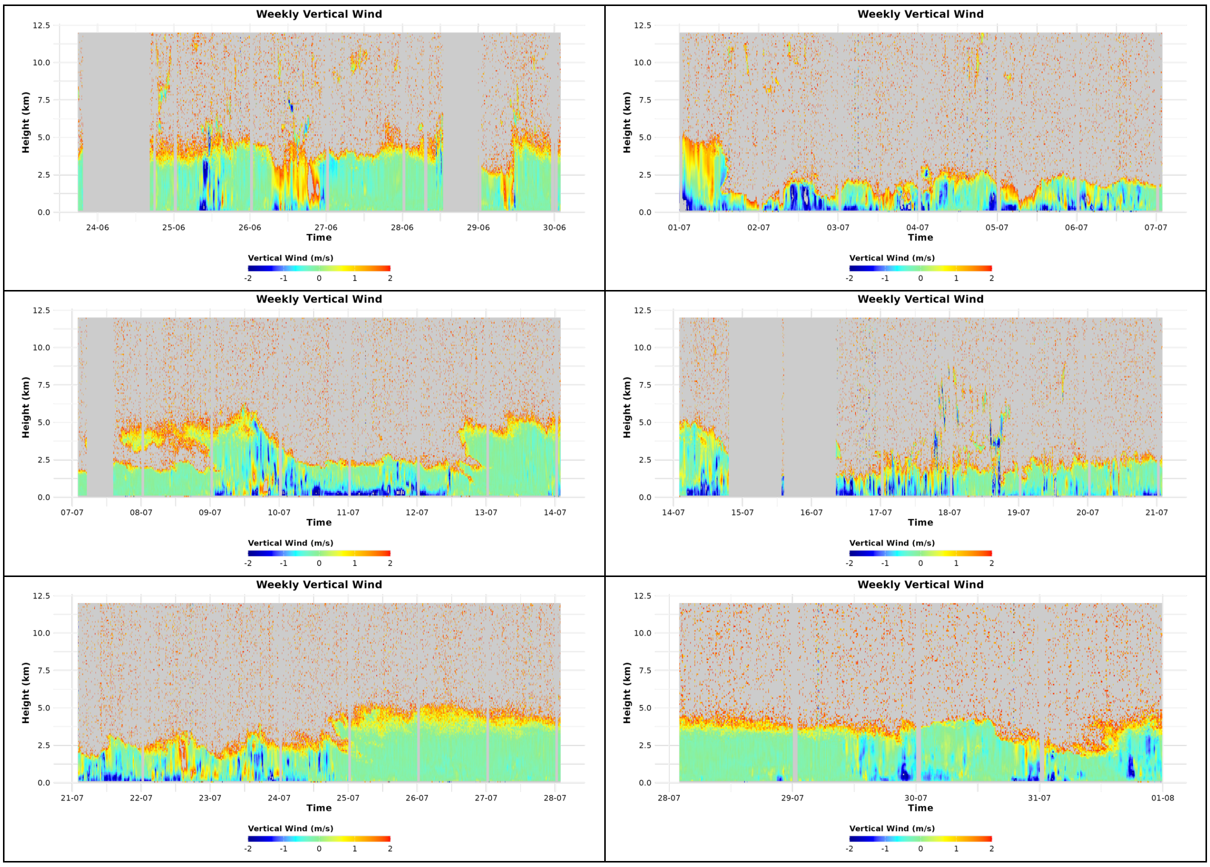

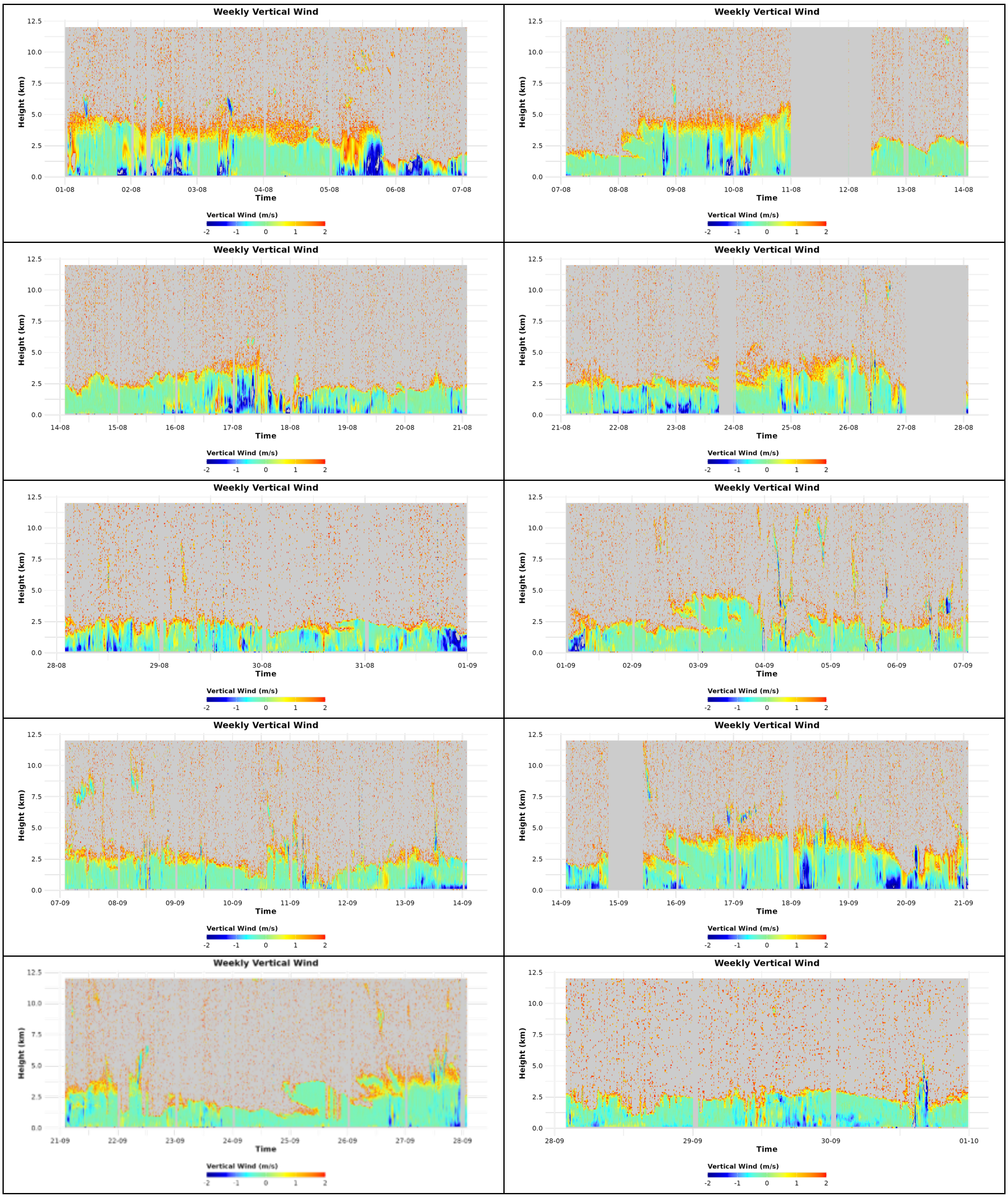

Figure S5: weekly time series of the vertical wind velocity retrieved from the lidar observations for the period June-September 2021. Panels are arranged sequentially, displaying the high-resolution measurements from the first week of the campaign in June through the final week in September.

The ERA5 grid point closest to the Soverato site was selected based on previous climatological studies (Madonna et al., 2022, https://doi.org/10.1029/2021JD035220), which demonstrated its representativeness for comparing tropospheric water vapor with upper-air observations. The study area, located at the center of the Mediterranean basin, exhibits complex orography, yet ERA5 at 0.25° resolution adequately captures the main atmospheric features in the free troposphere (300–500 hPa).

To assess spatial representativeness, bias and standard deviation of ERA5 relative humidity were evaluated across multiple nearby grid points over the study period at 300, 400, and 500 hPa. The nearest grid point shows a bias within ±1% RH for most of the domain, exceeding 2% only at 500 hPa over mountainous areas (Figure S6). Variability increases with distance but generally remains within 2–3% RH across the Tyrrhenian and Ionian coasts. These results indicate that alternative grid-point selections would have minimal impact on the analyses presented in the main manuscript. The additional plot included here further illustrates the spatial variability and supports the choice of the ERA5 grid point for comparison.

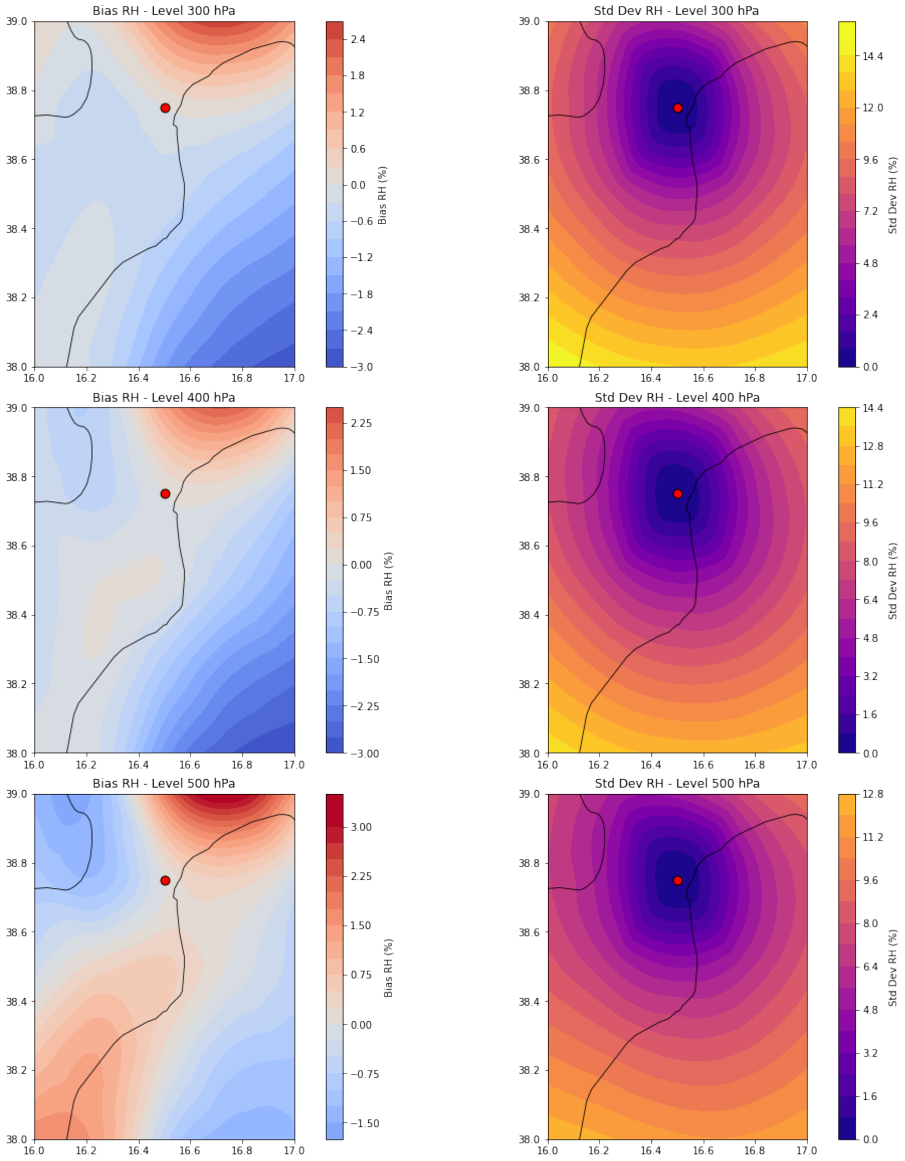

Figure S6: Bias (left panels) and standard deviation (right panels) between relative humidity for all ERA5 grid points within the domain 16-17°E longitude and 38-39°N latitude and the grid point closest to the Soverato site (red dot) at 300, 400, and 500 hPa.

An additional comparison to investigate the ERA5 dry bias is shown in Figure S7, where the RH measured by regular upper-air sounding balloons at Trapani Birgi RDS station (WIGOS ID: 0-20001-0-16429; 37.9142°N, 12.4914°E, 7.3 m asl) performed twice per day (00 and 12 UTC) are shown along with the corresponding ERA5 hourly time series from the nearest reanalysis grid point. Focusing on the mid-upper troposphere, the comparison reveals also in this case the presence of a dry bias in ERA5, which may exceed 10% RH, and, for the values between 300 hPa and 500 hPa in June, an overestimation of the RH between 300 and 400 hPa. More generally, the dry bias can be also clearly identified in all the highest RH values found in mid-upper troposphere, due to convection, water vapour fluxes or synoptic instability. It is noteworthy that ERA5 assimilates regular radiosounding data, which may have played a role in reducing the RH bias in Trapani compared to Soverato site, assuming the same profiles shown in right panel of Figure S7 have been part of the ERA5 input data stream.

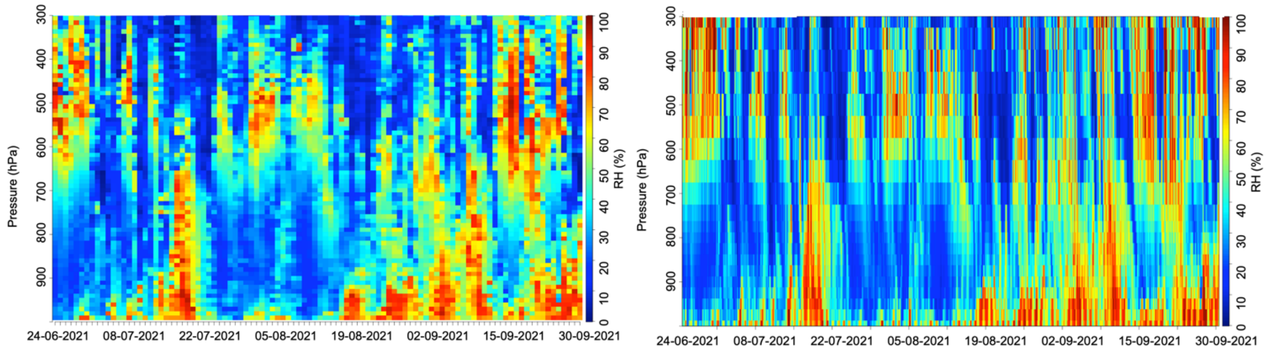

Figure S7: Comparison of relative humidity (RH) time series between Trapani Birgi radiosonde measurements (right panel) and ERA5 reanalysis (left panel) for the period 24 June 2021 to 30 September 2021. Radiosonde observations at Trapani Birgi RDS station (WIGOS ID: 0-20001-0-16429; 37.9142°N, 12.4914°E; 7.3 m asl) were performed twice daily at 00 and 12 UTC, while ERA5 data are provided at hourly resolution from the nearest reanalysis grid point.

Precipitable water vapour (PWV) estimated by the MWP is compared in Figure S8 with retrievals obtained from the co-located sun photometer and the ERA5 estimate for the nearest grid point to Soverato. The time sampling of the three datasets differs: hourly for ERA5, every 5 minutes for the MWP, and approximately every 15 minutes for the sun photometer. ERA5 data is the smoothest compared to the other instruments, both of which have a small field of view and during some periods, especially in the presence of thick clouds, sun photometer measurements are missing, as they are automatically filtered during processing by AERONET. Some MWP values are affected by precipitation in the first part of the campaign. During this period, when ERA5 overestimates clouds above 6 km, the MWP shows a higher value of water vapour than the sun photometer and ERA5, consistent with a greater fraction of the total atmospheric water being in the vapour phase.

Conversely, in the intermediate period of the campaign, the agreement between the MWP and the sun photometer is good, with ERA5 generally showing values smaller than MWP. It is important to note that, in addition to the pointed inconsistency in time resolution, ERA5 results is an average value over a grid box of 31 km space, not a point value, which may contribute to the obtained underestimation of the value due to sub-grid variability, particularly in convective scenarios. Overall, the three datasets tend to reproduce comparable patterns. The sun photometer, which measures not vertically but along the direction of the sun, represents a broader horizontal region of the atmosphere and it is sometimes in better agreement with ERA5, but most of the time is closer to the PWV from the MWP.

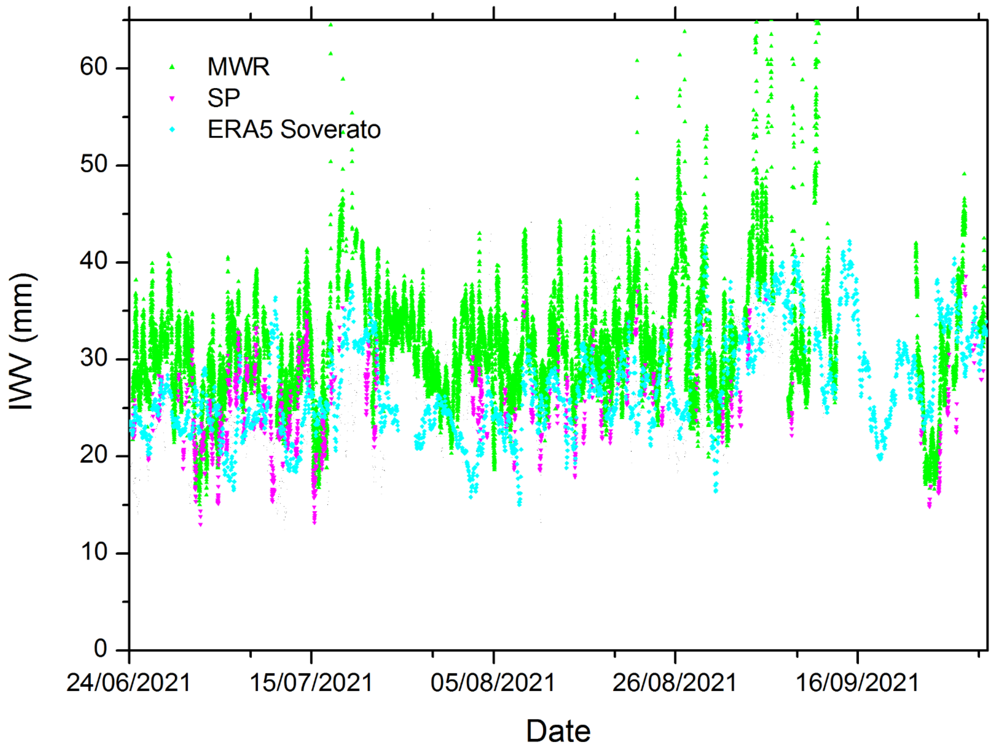

Figure S8: Integrated water vapor retrieved by the microwave radiometer (green), the sun photometer (magenta), and obtained from the ECMWF ERA5 reanalysis (cyan) from 24 June to 30 September 2021 at Soverato measurement site.

Figures S9 and S10 present the 500 hPa geopotential height and 2 m temperature fields (contour lines) from the ERA5 reanalysis for two key dates: July 5th and July 14th, both at 12 UTC. These figures illustrate the synoptic-scale atmospheric conditions influencing the region during the respective periods, providing context for the observed variability in water vapor transport and temperature patterns.

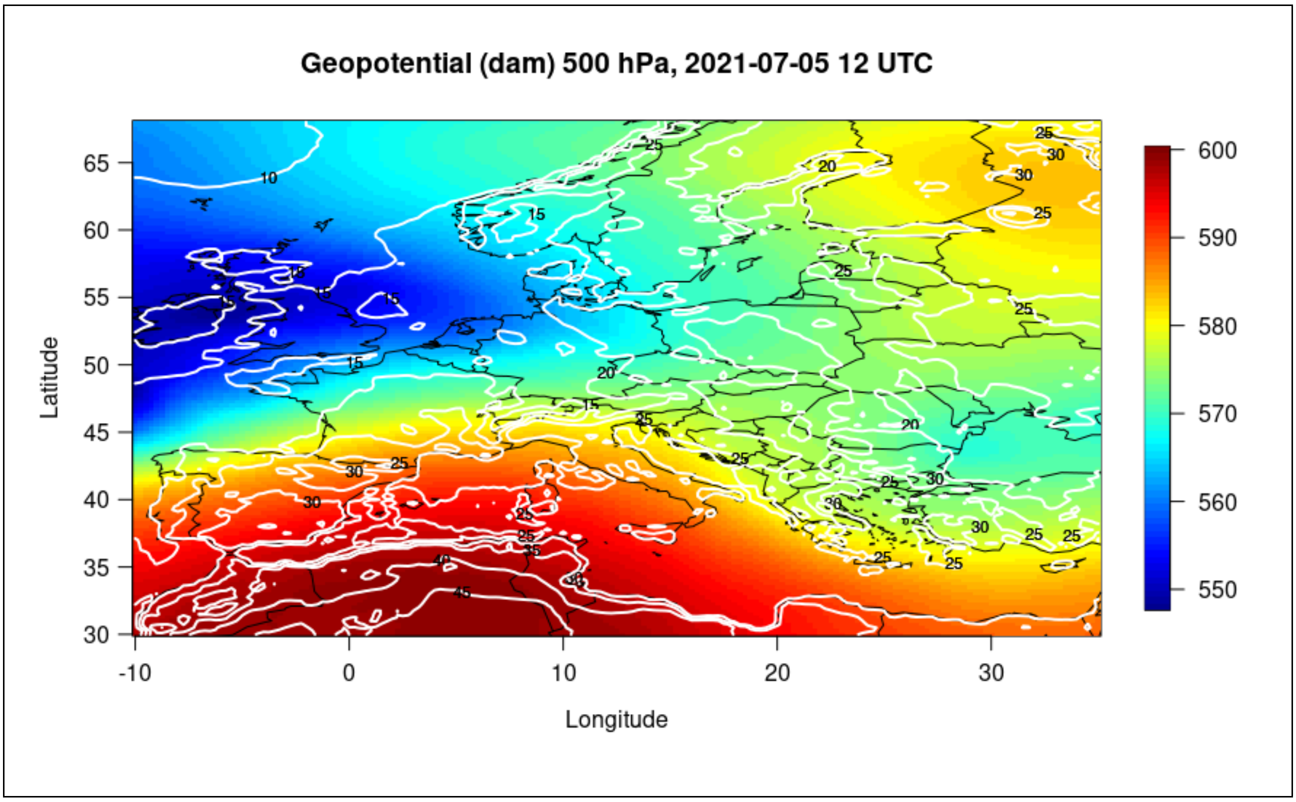

Figure S9: 500 hPa geopotential height and 2m temperature (contour lines) on July 5th from ERA5 reanalysis at 12 UTC.

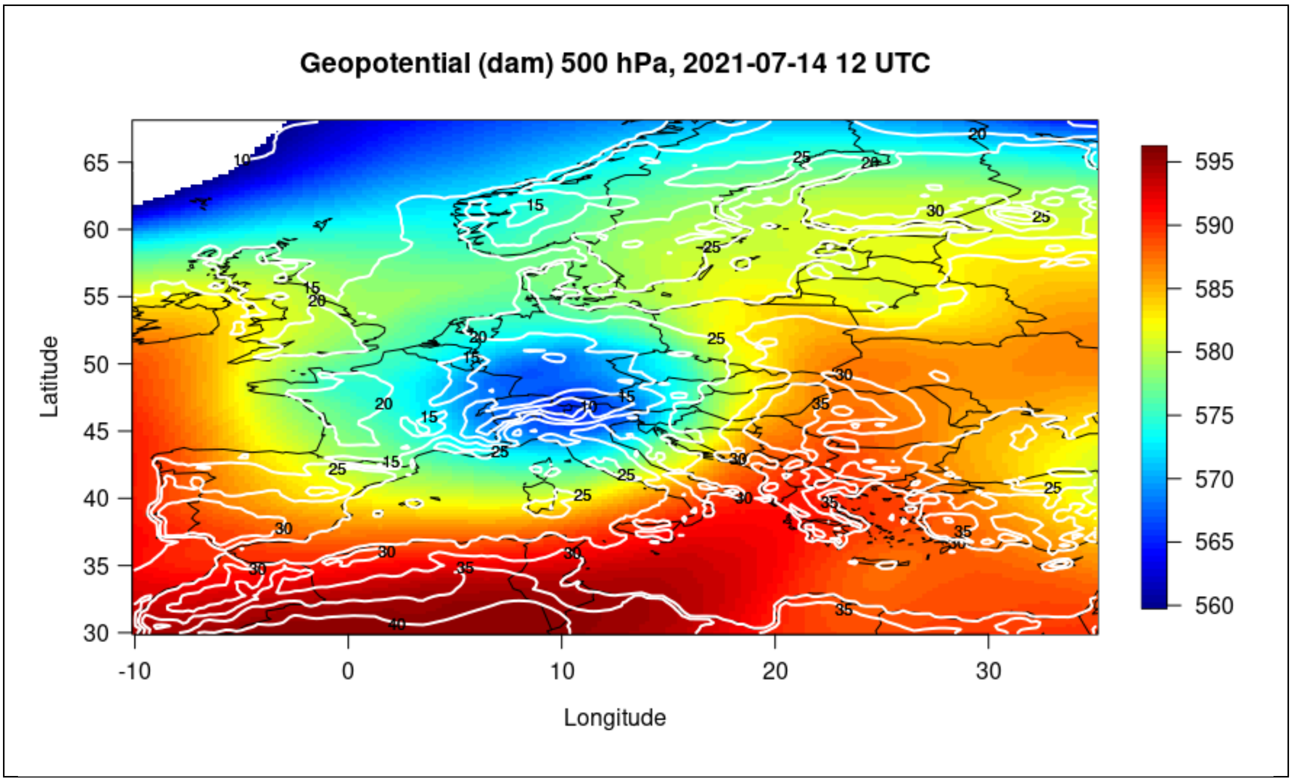

Figure S10: Same as Figure S9, but for July 14th at 12 UTC.

Backward HYSPLIT trajectories are also provided to identify the principal moisture source regions for the observations collected on 14 July (Figures S11) and 5 July 2021 (Figure S12). The trajectories are simulated at four altitude levels (3000, 4000, 5000 and 6000 m a.s.l.), with hourly initializations. Trajectories were driven by NCEP/NCAR reanalysis data, and the water vapor mixing ratio along each pathway was calculated from temperature and relative humidity fields. The water vapor mixing ratio along the 72-hour backward trajectories for the case of 14 July, as a function of time for each starting altitude, is shown in Figure S13. Figure S14 illustrates the same as Figure S13 for the case of 5 July.

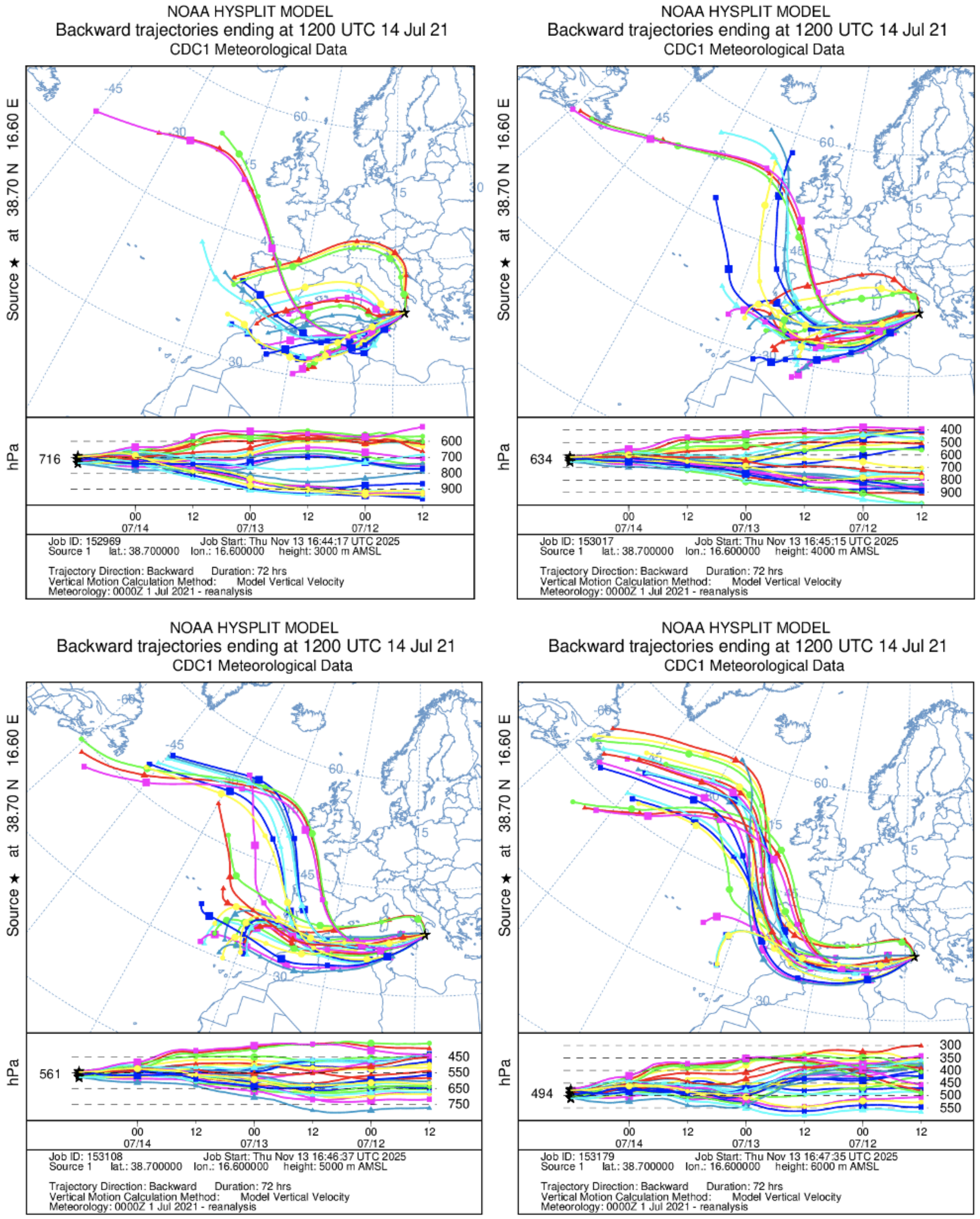

Figure S11: 72-hour backward HYSPLIT trajectories initialized hourly from Soverato at 3000, 4000, 5000, and 6000 m a.s.l.. The figure identifies the principal moisture source regions feeding the elevated water vapor content observed at Soverato on 14 July 2021.

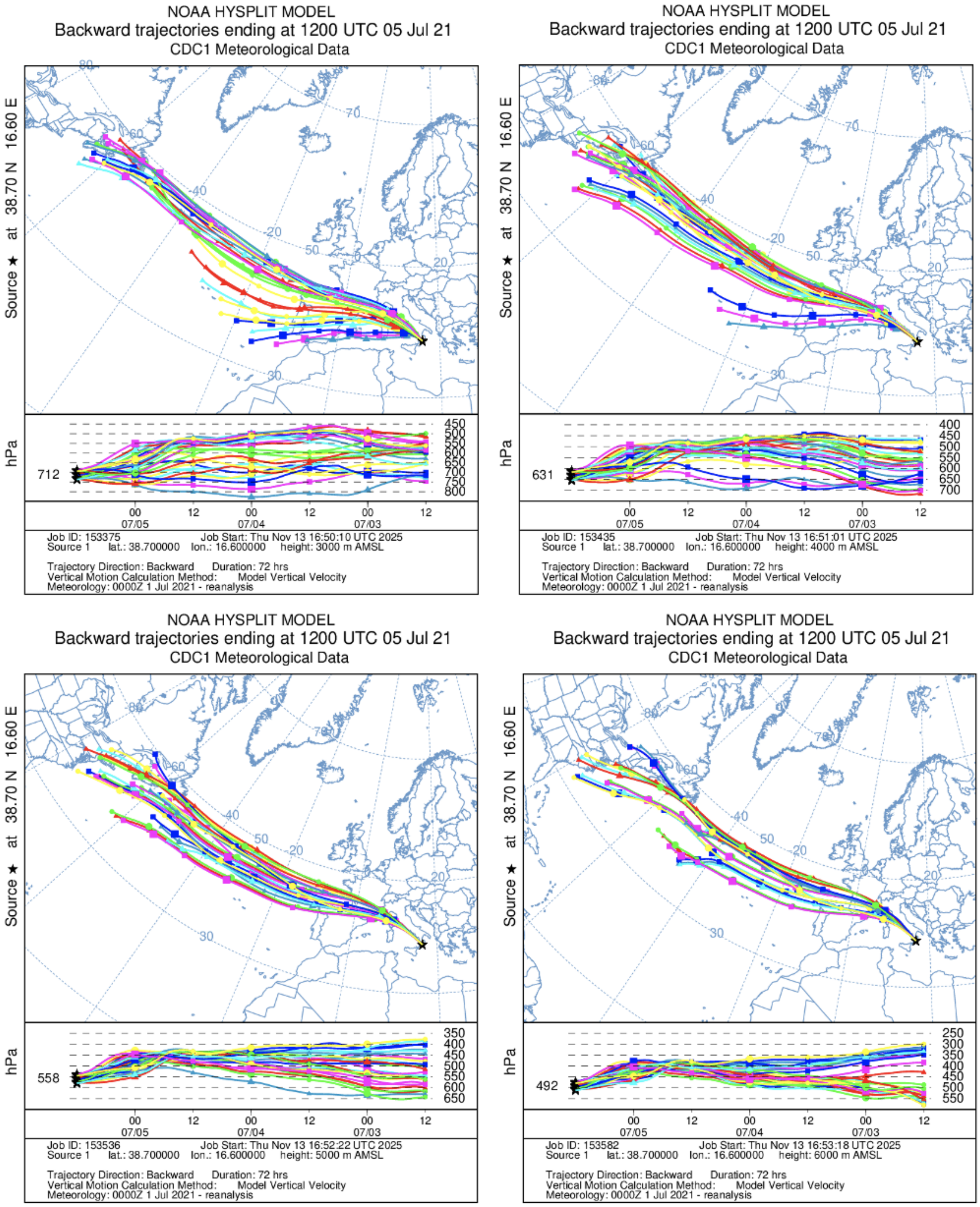

Figure S12: Same as Figure S11 for the 5 July 2021.

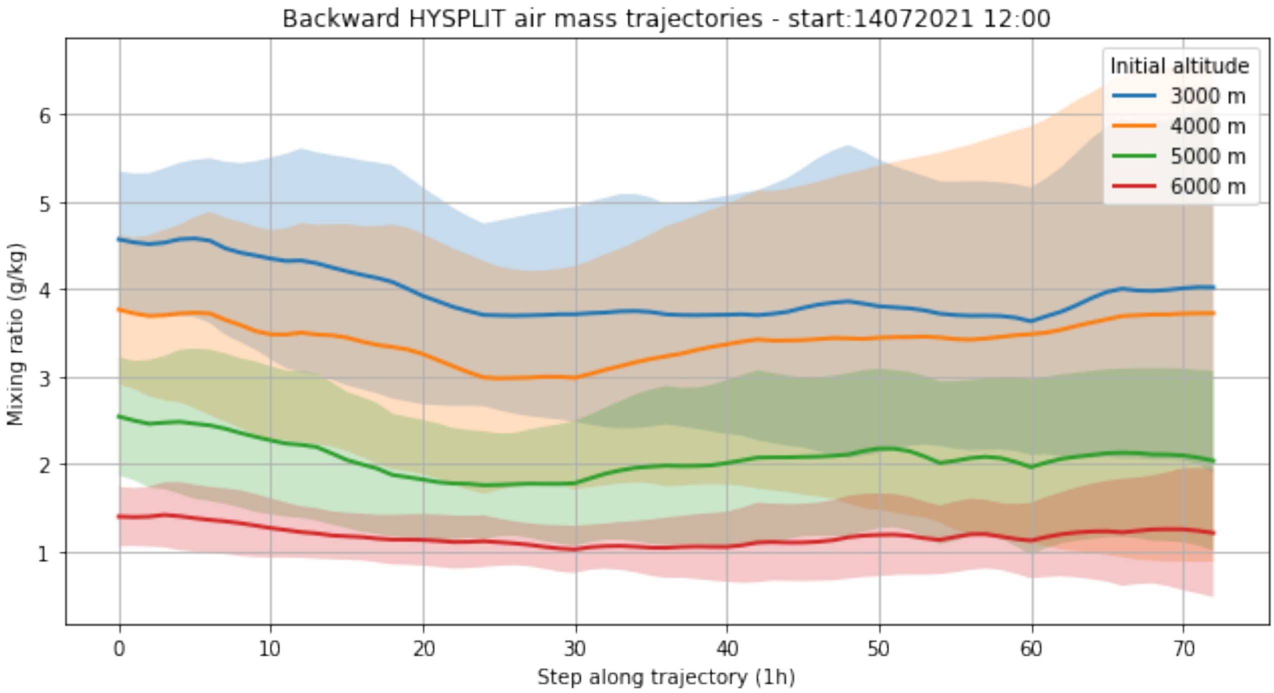

Figure S13: Water vapor mixing ratio (g kg^{-1}) along the 72-hour backward trajectories shown in Figure S11, expressed as a function of time for each starting altitude. The solid lines represent the mean mixing ratio of the air masses at the different altitude levels for each trajectory and time step, while the shaded areas indicate the corresponding standard deviation.

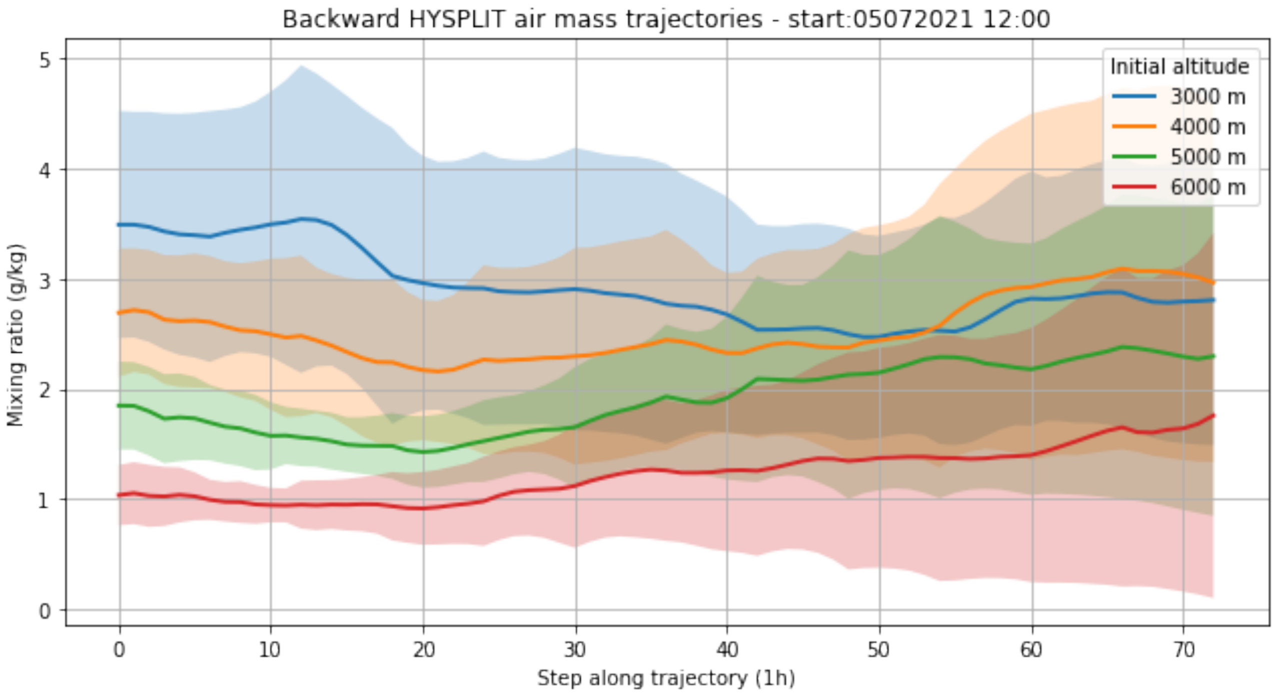

Figure S14: Same as Figure S13 for the 72-hour backward trajectories shown in Figure S12.

The influence of aerosol types in cloud formation during the campaign was examined using UV Raman lidar measurements. To this purpose, Figure S15 shows values of the 355 nm

lidar ratio of tropospheric aerosols, S, in the range between 1 and 6 km a.s.l. versus the corresponding values of the particle depolarization ratio, δ, measured with the UV polarization Raman lidar during the campaign. It illustrates the large variability of S for all measurements collected during the campaign, mainly at night, during measurement sessions averaging 2-3 hours. This variability highlights the different pure and mixed aerosol types observed on-site throughout the campaign. According to the literature (e.g. Müller et al., 2007; Burton et al., 2012; Groß et al., 2013; Papagiannopolous et al., 2018), the values of S and δ indicate the predominance of marine and continental aerosols, both pure and dust-contaminated, and a smaller amount of pure dust, which, from the range-resolved lidar measurements, is often located above 3 km. The analysis of the HYSPLIT air mass back trajectories reveals the main provenance from the Tyrrhenian and Adriatic seas for pure marine aerosol (trajectories can be retrieved from https://www.ready.noaa.gov/HYSPLIT.php at 1, 3, and 5 km agl using the "Model vertical velocity" and a total run of 96 hours). Contribution from biomass burning aerosol was also present, mostly during the first part of the campaign, identified through photos and sky imagers' data collected at the site.

In terms of average aerosol size distribution, as retrieved using the sun photometer (SP), the volume concentrations and effective radius of aerosols estimated as monthly averages for July and September 2021 only has been compared (Table S2), because no inversion data from the SP data available in August 2021 (due to instrumental issues), hence no estimation of the aerosol size distribution parameters. Results reveal an increase in the volume concentration for the coarse fraction in September, although the dominant mode remains coarse, even in July. Properties of the fine particles are quite similar for both months, with a wider distribution in September, except for the coarse mode. The dominance of the aerosol coarse fraction, along with evidence for the predominant contribution of marine aerosols and desert dust, suggests that the coarse particles observed during the summer of 2021 in Soverato played a key role. Under atmospheric conditions, typically dominated by persistent high pressures over South Italy, these particles contributed to a reduced likelihood of warm cloud formation at the measurement site. Despite the presence of ice nuclei at the site, of both mineral and marine nature, there was also a scarcity of high clouds during the campaign that, beyond the subsidence related to the high-pressure, was due to the occurrence of heat waves, often generating strong inversions in the temperature profiles acting as a barrier to vertical air movement, preventing the moist air at lower levels from rising to higher altitudes where cirrus clouds could form.

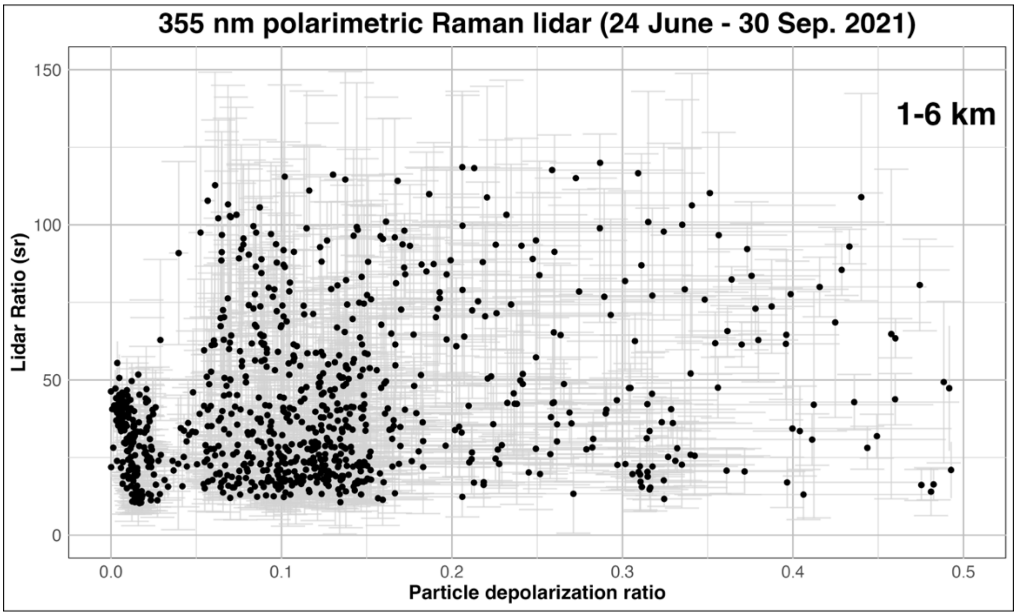

Figure S15: values of the 355 nm lidar ratio of tropospheric aerosols in the range between 1 and 6 km a.s.l. versus the corresponding values of the particle depolarization ratio measured with the UV polarization Raman lidar during the campaign at Soverato from 24th June to 30th September 2021. The gray horizontal and vertical bars are representative of the corresponding statistical uncertainties of the plotted quantities. Only values with less than 40% relative uncertainty on the lidar ratios have been sleeked.

Finally, Table S1 shows the regional climatology of CAPE and CIN values in the period 1994-2020, for the Calabria region. Key historical percentiles (10th, 25th, 50th, 75th, 90th) are reported for each summer month (June–September). Considering the values observed at the Soverato site, during the campaign period, and comparing them with the regional climatology, the following summary can be provided.

CAPE:

- June values (525 J kg^{-1}) lie above the 75th percentile of the climatological distribution, while July values (506 J kg^{-1}) fall between the median and the 75th percentile.
- August 2021 exhibits a marked increase (~795 J kg^{-1}), exceeding the climatological median and approaching the 75th percentile, indicating moderately enhanced convective energy.
- September values (~570 J kg^{-1}) are slightly elevated relative to the median and fall within the 75th–90th percentile range.

CIN:

- CIN values in June and July 2021 (~163–197 J kg^{-1}) are close to, or slightly above, the 25th percentile, suggesting typical convective inhibition conditions.

- August 2021 shows elevated CIN (~470 J kg^{-1}), above the median and approaching the 75th percentile, consistent with temporarily stronger atmospheric stability.
- September values (~268 J kg^{-1}) are slightly above the median but remain well within the climatological range.

Overall, convective parameters observed at Soverato region in 2021 are moderately elevated compared to the historical record. The concurrent peaks in CAPE and CIN during August stand out as the most distinctive feature of the summer, reflecting enhanced convective potential alongside increased inhibition. Nevertheless, all values remain within the 10th–90th percentile range of the regional climatology, indicating that these anomalies, while noteworthy, do not represent extreme conditions.

Table S1: Percentiles (10th, 25th, 50th, 75th, 90th) of the regional climatology of CAPE and CIN values for the Soverato region in the period 1994-2020. The climatology has been estimated over the southern part of the continental Italy (i.e. Calabria region).

CAPE

month	Median 2021	p10	p25	p50	p75	p90
6	525	0	0	50	428	1146
7	506	0	10	180	750	1411
8	794	0	52.5	414	1251	2107
9	570	0	2.25	89	518	1165

CIN

month	Median 2021	p10	p25	p50	p75	p90
6	162	63	161	349	553	737
7	197	75	161	338	574	760
8	470	77	173	357	583	778
9	267	20	66	175	417	666

Month	2021-JUL	2021-SEP
VolC-T	0.10	0.15
REff-T	0.47	0.72
VMR-T	1.16	1.66
Std-T	1.32	1.06
VolC-F	0.02	0.02
REff-F	0.14	0.12
VMR-F	0.15	0.14
Std-F	0.47	0.57
VolC-C	0.08	0.14
REff-C	1.87	1.77
VMR-C	2.38	2.20
Std-C	0.68	0.65

Table S2: Monthly average values of the total volume concentration (VolC-T), fine mode concentration (VolC-F), and coarse mode concentration (VolC-C) of the aerosol size distribution as estimated by the sun photometer during the campaign at Soverato for the months of July and September 2021. REff, Std and VMR indicate the corresponding particle effective radius, the standard deviation of the effective radius and the volume mean radius. All the quantities are reported in μm. Data have been processed using the AERONET microphysical retrieval (https://aeronet.gsfc.nasa.gov/new_web/Documents/Inversion_products_for_V3.pdf).

References

Burton, S. P., Ferrare, R. A., Hostetler, C. A., Hair, J. W., Rogers, R. R., Obland, M. D., Butler, C. F., Cook, A. L., Harper, D. B., and Froyd, K. D.: Aerosol classification using airborne High Spectral Resolution Lidar measurements – methodology and examples, Atmos. Meas. Tech., 5, 73–98, https://doi.org/10.5194/amt-5-73-2012, 2012.

Dessler AE, Zhang Z, Yang P. Water-vapor climate feedback inferred from climate fluctuations, 2003–2008. Geophys. Res. Lett. 2008;35:20. doi: 10.1029/2008GL035333.

Groß, S., Esselborn, M., Weinzierl, B., Wirth, M., Fix, A., and Petzold, A.: Aerosol classification by airborne high spectral resolution lidar observations, Atmos. Chem. Phys., 13, 2487–2505, https://doi.org/10.5194/acp-13-2487-2013, 2013.

Müller, D., Heinold, B., Tesche, M., Tegen, I., Althausen, D., Alados-Arboledas, L., Amiridis, V., Amodeo, A., Ansmann, A., Balis, D., Comeron, A., D'Amico, G., Gerasopoulos, E., Guerrero-Rascado, J. L., Freudenthaler, V., Giannakaki, E., Heese, B., Iarlori, M., Knippertz, P., Mamouri, R. E., Mona, L., Papayannis, A., Pappalardo, G., Perrone, R.-M., Pisani, G., Rizi, V., Sicard, M., Spinelli, N., Tafuro, A., and Wiegner, M.: EARLINET observations of the 14–22-May long-range dust transport event during SAMUM 2006: validation of results from dust transport modelling, Tellus B, 61, 325–339, https://doi.org/10.1111/j.1600-0889.2008.00400.x.

Papagiannopoulos, N., Mona, L., Amodeo, A., D'Amico, G., Gumà Claramunt, P., Pappalardo, G., Alados-Arboledas, L., Guerrero-Rascado, J. L., Amiridis, V., Kokkalis, P., Apituley, A., Baars, H., Schwarz, A., Wandinger, U., Binietoglou, I., Nicolae, D., Bortoli, D., Comerón, A., Rodríguez-Gómez, A., Sicard, M., Papayannis, A., and Wiegner, M.: An automatic observation-based aerosol typing

method for EARLINET, Atmos. Chem. Phys., 18, 15879–15901, https://doi.org/10.5194/acp-18-15879-2018, 2018.
