# Supplementary figures and images for: Water vapor transport observed at a coastal Mediterranean site during the summer of 2021 and compared with ERA5
Source: Sci Rep. 2026 Feb 14;16:9105. doi: 10.1038/s41598-026-36040-0 (PMC12996470; doi:10.1038/s41598-026-36040-0)

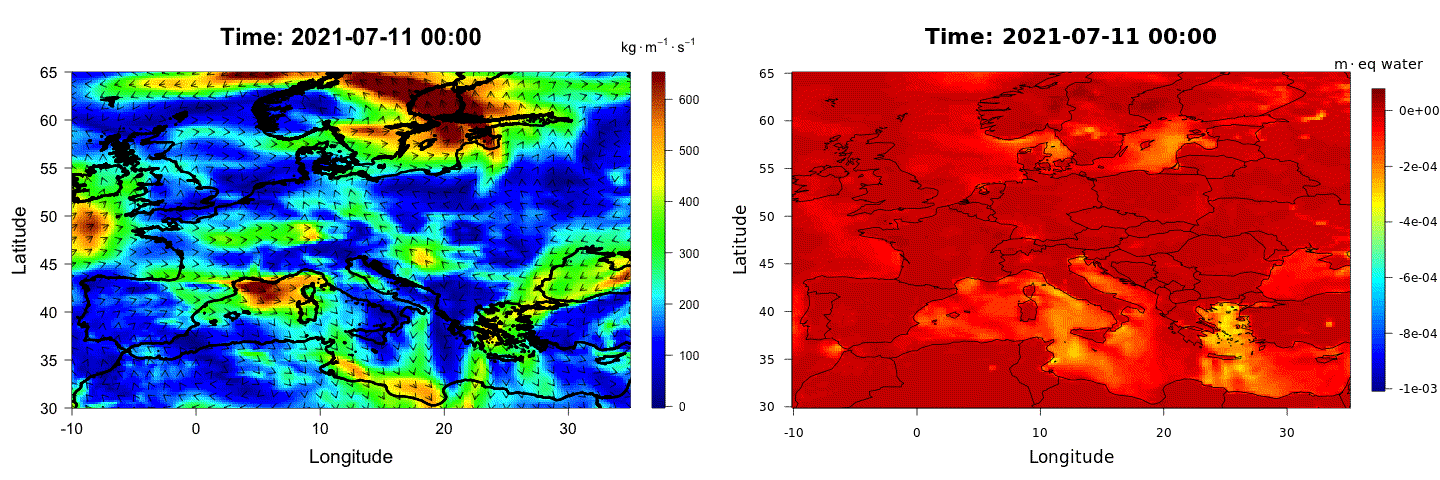

Supplement: Supplementary file 1 — Supplementary Material 1 [file 41598_2026_36040_MOESM1_ESM.zip › Supplement_material/combined_animation_figure6_700_1000..gif]

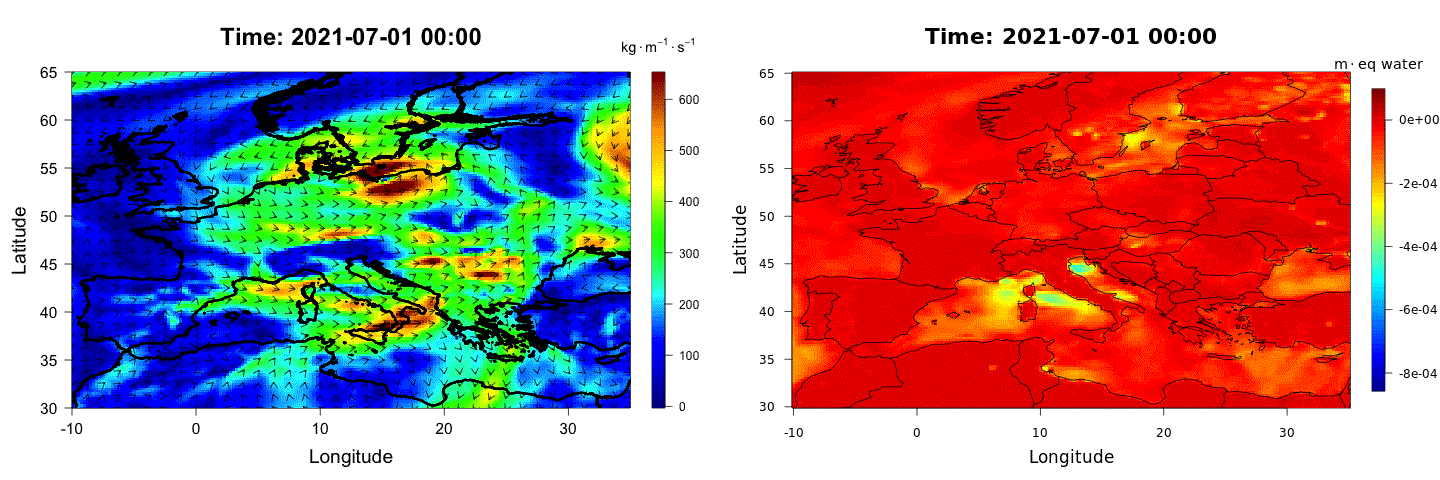

Supplement: Supplementary file 1 — Supplementary Material 1 [file 41598_2026_36040_MOESM1_ESM.zip › Supplement_material/combined_animation_figure5_700_1000.gif]

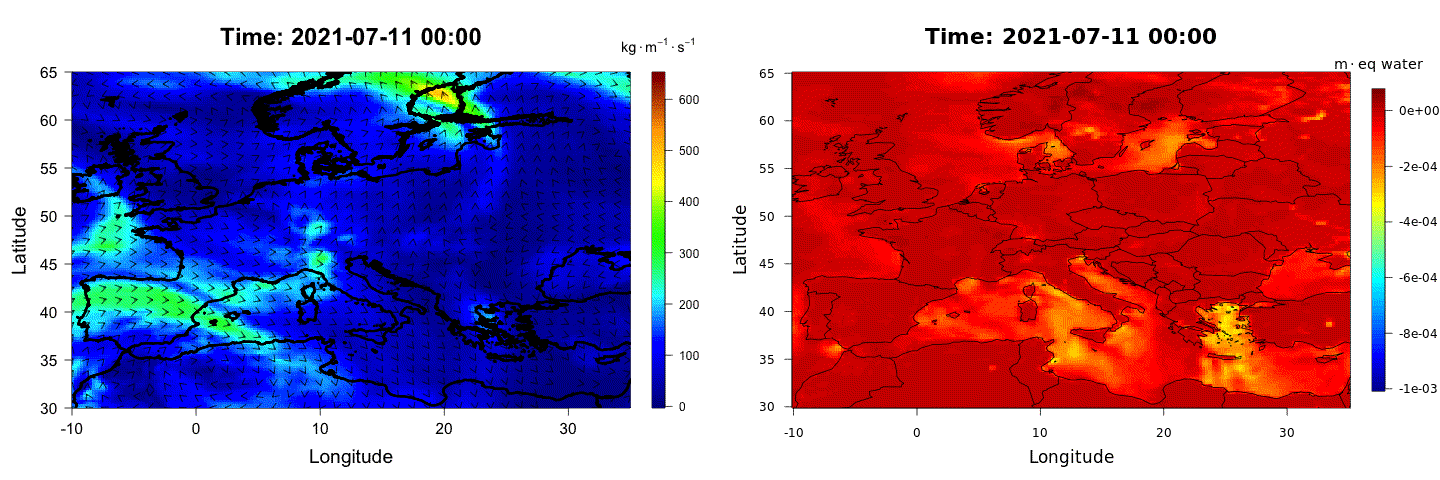

Supplement: Supplementary file 1 — Supplementary Material 1 [file 41598_2026_36040_MOESM1_ESM.zip › Supplement_material/combined_animation_figure6_300_700.gif]

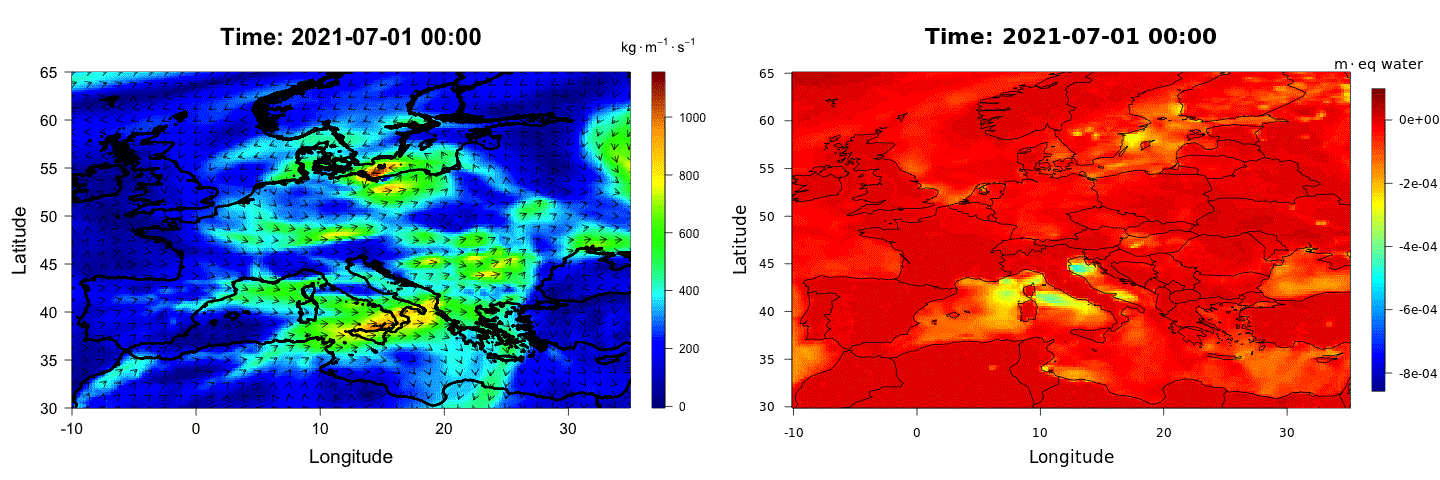

Supplement: Supplementary file 1 — Supplementary Material 1 [file 41598_2026_36040_MOESM1_ESM.zip › Supplement_material/combined_animation_figure5_300_1000.gif]

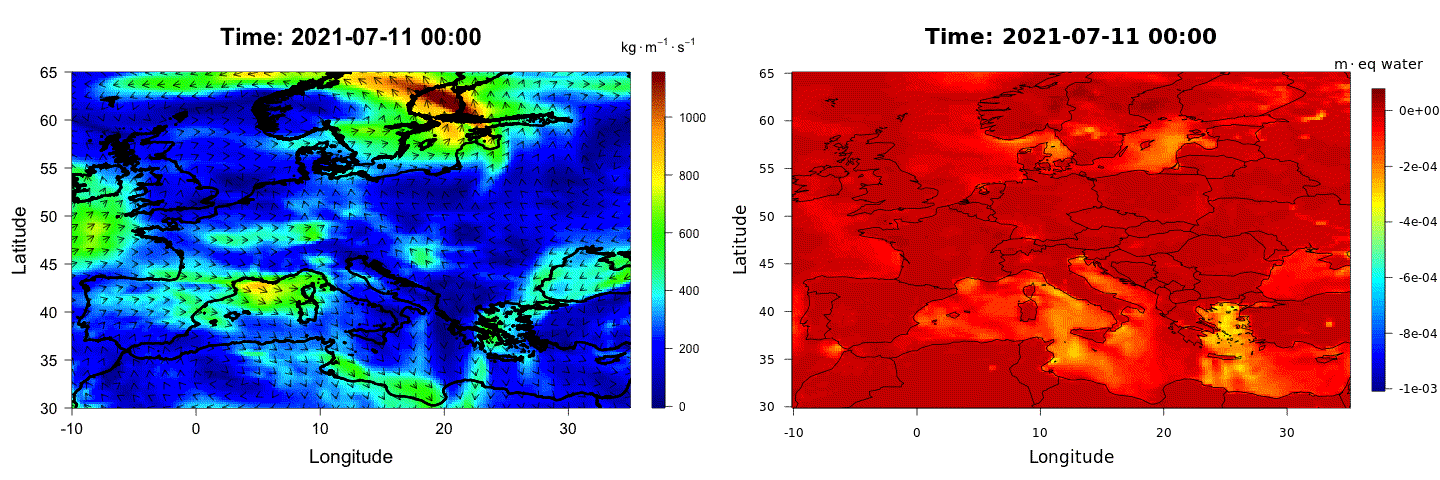

Supplement: Supplementary file 1 — Supplementary Material 1 [file 41598_2026_36040_MOESM1_ESM.zip › Supplement_material/combined_animation_figure6_300_1000..gif]

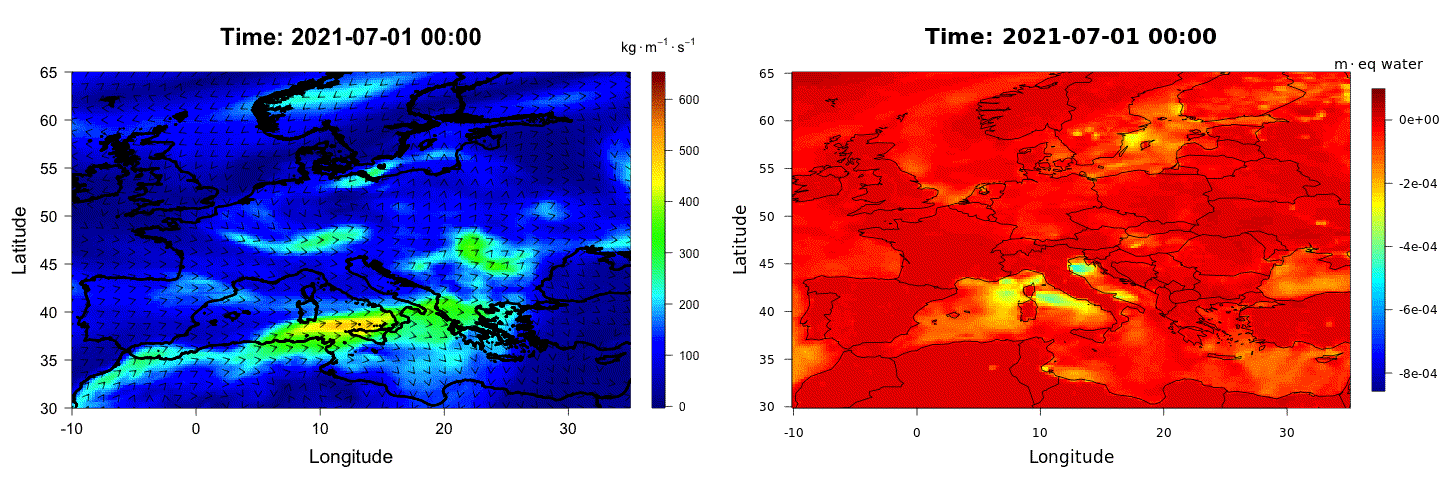

Supplement: Supplementary file 1 — Supplementary Material 1 [file 41598_2026_36040_MOESM1_ESM.zip › Supplement_material/combined_animation_figure5_300_700.gif]
